# A first checklist of macrofungi for South Africa

**DOI:** 10.3897/mycokeys.63.36566

**Published:** 2020-02-05

**Authors:** Tonjock Rosemary Kinge, Gary Goldman, Adriaana Jacobs, George Gatere Ndiritu, Marieka Gryzenhout

**Affiliations:** 1 Department of Genetics, University of the Free State, Bloemfontein, PO Box 339, Bloemfontein 9300, Republic of South Africa University of the Free State Bloemfontein South Africa; 2 Department of Biological Sciences, Faculty of Science, University of Bamenda, P.O. Box 39, Bambili, North West Region, Cameroon University of Bamenda Bambili Cambodia; 3 MushroomFundi, Cape Town, South Africa MushroomFundi Cape Town South Africa; 4 National Collection of Fungi, Mycology Unit, Plant Health and Protection, Agricultural Research Council, Pretoria, South Africa National Collection of Fungi Pretoria South Africa; 5 School of Natural Resources and Environmental Studies, Karatina University, P.O. Box 1957, Karatina 10101, Kenya Karatina University Karatina Kenya

**Keywords:** biodiversity, conservation, macrofungi, Myxomycetes, slimemolds, South Africa, species lists

## Abstract

Macrofungi are considered as organisms that form large fruiting bodies above or below the ground that are visible without the aid of a microscope. These fungi include most basidiomycetes and a small number of ascomycetes. Macrofungi have different ecological roles and uses, where some are edible, medicinal, poisonous, decomposers, saprotrophs, predators and pathogens, and they are often used for innovative biotechnological, medicinal and ecological applications. However, comprehensive checklists, and compilations on the diversity and distribution of mushrooms are lacking for South Africa, which makes regulation, conservation and inclusion in national biodiversity initiatives difficult. In this review, we compiled a checklist of macrofungi for the first time (excluding lichens). Data were compiled based on available literature in journals, books and fungorium records from the National Collection of Fungi. Even if the list is not complete due to numerous unreported species present in South Africa, it still represents an overview of the current knowledge of the macromycetes of South Africa. The list of names enables the assessment of gaps in collections and knowledge on the fungal biodiversity of South Africa, and downstream applications such as defining residency status of species. It provides a foundation for new names to be added in future towards developing a list that will be as complete as possible, and that can be used by a wide audience including scientists, authorities and the public.

## Introduction

Macrofungi are fungi that form large fructifications visible without the aid of the microscope and include representatives from the *Basidiomycota* and *Ascomycota* (Roda 2010; Servi et al. 2010). Common names used to refer to these fungi include mushrooms, toadstools, cup fungi, gilled fungi, jelly fungi, coral fungi, stink fungi, bracket fungi, polypores, puffballs, earth starts, truffles, and birds nest fungi (Egbe et al. 2013) and illustrates the visibility of these fungi to the public. Ecologically, macrofungi can be grouped as saprobes, parasites and symbiotic species (for instance mycorrhiza). Most terrestrial macrofungi are saprobes or mycorrhizal symbionts, but some are pathogens of plants or fungi, while those fruiting on woody substrates are usually either saprobes or plant pathogens (Mueller et al. 2007; Maria and Tzenka 2014).

Many macrofungi are edible and rich sources of carbohydrates, proteins, vitamins, and minerals for humans (Ananbeh 2003; Gençcelep et al. 2009). They can be naturally harvested or cultivated commercially. For rural communities they serve as a source of protein and income, especially for women. Macrofungi have great bio-exploitation potential in medicine or industry such as in the production of penicillin, lovastatin, and other globally significant medicines, and they remain an untapped resource with enormous industrial potential (Hyde et al. 2019). Mushrooms and other types of macrofungi can grow on decayed organic matters rich in lignin, cellulose, and other complicated carbohydrates, breaking them down for other uses or for bioremediation purposes (Kulshrestha et al. 2014). Modern pharmacological research confirms that large parts of traditional knowledge regarding the medicinal effects of macrofungi are due to proven antifungal, antibacterial, antioxidant, antiviral or other medicinal properties, besides being used as functional foods (Wani et al. 2010). For instance, some of the best known substances present in fungi showing pharmacological properties (especially anticancer and immunological) are polysaccharides (Wasser 2002; Mordali et al. 2007; Zhang et al. 2007; Hyde et al. 2019). Polysaccharides or polysaccharide-protein complexes present in fungi have gained the attention of researchers because it is believed that they can inhibit tumor growth and boost the immune system of the organism. They can enhance host defensive potential or represent biological response modifiers (Leung et al. 2006; Mordali et al. 2007). However, regulation of fungal bio-exploration and research in South Africa is hampered by the absence of biodiversity knowledge.

The fruiting bodies of slime molds or myxomycetes are occasionally observed together with those of macrofungi. The first species was described in 1654 by naturalist Thomas Panckow, who thought it was a species of fungi because of its resemblance to puffballs (Martin and Alexopoulos 1969). Slime molds have two major stages in their lifecycles: a mobile trophic (feeding) and a static fruiting body (reproductive) stage. Modern classifications place them in the Mycetozoan group of Amoebozoa (Baudalf 2008; Fiore-Donno et al. 2010). As bacterivores, slime molds are major components of decomposition and nutrient cycles where they enhance release of nutrients tied up in the microbial biomass (Stephenson and Feest 2015). It is estimated that myxomycete amoebas alone represent more than 50% of the total amoebae for some agricultural soils (Feest and Campbell 1986). Recent studies suggest that more attention should be placed on the use of slime molds as indicators of soil quality.

A small percentage of the 2.2 to 3.8 million species of fungi estimated in the world are currently described and these are mostly in temperate regions (Hawksworth and Lücking 2017). The tropical regions with the highest fungal diversity have not been fully explored (Hawksworth 2001). The reasons for this disparity, even in First World countries, are taxonomic obstacles that are worsened by a paucity of trained mycologists and especially systematists. The low number of published, rigorous, long-term studies on fungal biodiversity also prevents conclusive answers (Mueller et al. 2007). Not even basic questions, such as those related to the number of macrofungal or slime mold species at a specific location, or whether such diversity is greater in one type of forest than in another, can often be answered.

Despite the importance of macrofungi, information on their diversity is scanty, especially in Africa (Osarenkhoe et al. 2014). Thus, due to the lack of human capacities, national monographs of biodiversity in many African countries rarely encompass fungi. This leads to an unfortunate bias in the complete assessment of biodiversity, the unawareness by the public and decision makers of fungi as important components of ecosystem functioning, and frustration from end users at the lack of information (Gryzenhout et al. 2012). Regulation of fungal natural resources and quarantine is thus severely impeded by the lack of lists and information readily available. Not surprisingly, the fungal biodiversity in southern Africa has been relatively poorly studied to date, and no host has been thoroughly treated (Crous et al. 2006; Gryzenhout et al. 2010, 2012). A working checklist will be greatly beneficial to illustrate strengths and gaps in our fungal biodiversity knowledge in South Africa, and will be useful for regulatory authorities.

To address the lack of basic information for macrofungi in South Africa, the aim of this review was to compile a macrofungal and slime mold names list based on current knowledge and resources. We defined macrofungi as having spore-bearing structures visible to the naked eye (mushrooms, brackets, puffballs, false-truffles, cup fungi, etc.). Since slime molds are also readily observed by the public and perceived as fungi (although they do not reside in the kingdom of Fungi), known slime molds from South Africa were also included. Lichens (structures formed by fungi living in close association with algae and cyanobacteria) were excluded from this review because they represent another ill-studied group without solid supportive capacity, but lichen species should be added in future.

## Materials and methods

The species list was compiled from journal and book publications, and national fungorium records. It is not based on field observations. It is hosted on the website www.themycologyblog.com, which is live and can continuously be refined, expanded and updated. The species list is incorporated by the online resource Cybertruffle’s (http://www.cybertruffle.org.uk/) and the database of the National Collection of Fungi of South Africa (http://www.arc.agric.za/arc-ppri/Pages/Biosystematics/Mycology%20Unit%20(Fungi)/Mycology-National-Collection-of-Fungi.aspx).

## Results

The macrofungal checklist compiled in this review (Table 1) presents the first national list for macrofungi and slime molds in South Africa. It includes macrofungal and slime mold species names from previous field guides, other publications, as well as names obtained from the National Collection of Fungi’s PREM fungorium (based on 3597 records), hosted by the Plant Health and Protection (http://www.arc.agric.za/arc-ppri/Pages/ARC-PPRI-Homepage), Agricultural Research Council, South Africa (Table 1). Myxomycete records include 107 species. In total, the South African checklist presented here includes 1160 species, 307 genera and 95 families.

**Table 1.** Checklist of macrofungi and myxomycetes from South Africa.

Kingdom	Phylum	Class	Order	Family	Genus	Species	Authority	Fungarium	Field guides	Previous publications (if not in field guides)
Fungi	Ascomycota	Leotiomycetes	Helotiales	Chlorociboriaceae	* Chlorociboria *	* aeruginosa *	(Nyl.) Kanouse ex C.S. Ramamurthi, Korf & L.R. Batra		Yes	
		Pezizomycetes	Pezizales	Helvellaceae	* Paxina *	* leucomelas *	(Pers.) Kuntze		Yes	
					* Helvella *	* crispa *	(Scop.) Fr.		Yes	
						* lacunosa *	Afzel.		Yes	
				Morchellaceae	* Morchella *	* conica *	Krombh.	PREM	Yes	
						* elata *	Fr.		Yes	
						* hybrida *	Pers.	PREM		
				Pezizaceae	* Kalaharituber *	* pfeilii *	(Henn.) Trappe & Kagan-Zur		Yes	
					* Peziza *	* ammophila *	Saut.		Yes	
						* dehnii *	Rabenh.	PREM		
						* hortensis *	P. Crouan & H. Crouan	PREM		
						* macropus *	Schumach.	PREM		
						* nilgherrensis *	Cooke	PREM		
						* ostracoderma *	Korf	PREM		
						* repanda *	Pers.	PREM		
						* silvestris *	(Boud.) Sacc. & Traverso	PREM		
						* spissa *	Berk.	PREM		
						* subrepanda *	Cooke & W. Phillips	PREM		
						* vesiculosa *	Bull.	PREM		
					* Pseudohelotium *	* pineti *	(Batsch) Fuckel	PREM		
					* Terfezia *	* austroafricana *	Marasas & Trappe			Marasas and Trappe 1973
						* bourdieri *	Chatin		Yes	
						* claveryi *	Chatin		Yes	
				Pyrenomataceae	* Anthracobia *	* melaloma *	(Alb. & Schwein.) Arnould		Yes	
					* Isaria *	* psychidae *	Pole-Evans	PREM		
						* sinclairii *	(Berk.) Lloyd		Yes	
					* Scutellinia *	* badio-berbis *	(Berk. ex Cooke) Kuntze.	PREM		
						* margaritacea *	(Berk. ex Cooke) Kuntze.	PREM		
						* phlyctispora *	(Lepr. and Mont.)	PREM		
						* scutellata *	(L.) Lamb.	PREM	Yes	
				Rhizinaceae	* Rhizina *	* undulata *	Fr.		Yes	
				Sarcoscyphaceae	* Phillipsia *	* kermesina *	Kalchbr. & Cooke			Kalchbrenner and Cooke 1880
				Tuberaceae	* Tuber *	* aestivum *	(Wulfen) Spreng	PREM		
						* melanosporum *	Vittad.		Yes	
					* Choiromyces *	* echinulatus *	Trappe & Marasas			Trappe and Marasas 1973
		Sordariomycetes	Hypocreales	Cordycipitaceae	* Cordyceps *	* velutipes *	Massee			Massee 1895
Fungi	Ascomycota	Sordariomycetes	Xylariales	Xylariaceae	* Daldinia *	* concentrica *	(Bolton) Ces. & De Not.		Yes	
					* Poronia *	* oedipus *	(Mont.) Mont.		Yes	
					* Xylaria *	* longipes *	Nitschke		Yes	
						* hypoxylon *	L. (Grev.)		Yes	
						* polymorpha *	(Pers.) Grev.		Yes	
						* schreuderiana *	Van der Byl			Van der Byl 1932
						* stilboidea *	Kalchbr. & Cooke			Kalchbrenner and Cooke 1880
					* Penzigia *	* verrucosa *	Mill			Miller 1942
	Basidiomycota	Agaricomycetes	Agaricales	Agaricaceae	* Agaricus *	* actiniceps *	Kalchbr. & Cooke			Kalchbrenner and Cooke 1881
						* abruptibulbus *	Peck	PREM		Van der Westhuizen and Eicker 1988
						* alboargillascens *	(A. Pearson) Bon			
						* alveolatus *	Kalchbr.			Kalchbrenner 1881
						* arvensis *	Schaeff.	PREM	Yes	
						* augustus *	Fr.	PREM	Yes	
						* aures *	(Massee) F.M. Bailey	PREM		
						* bisporus *	(J.E. Lange) Imbach	PREM	Yes	
						* bitorquis *	(Quél.) Sacc.	PREM	Yes	
						* caliginosus *	Jungh.	PREM		
						* campestris *	L.	PREM	Yes	
						* chortophilus *	Berk.			Berkeley 1843
						* comtulus *	Ces. ex Mussat	PREM	Yes	
						* crocopeplus *	Berk. & Broome		Yes	
						* diminutivus *	Peck		Yes	
						* episphaeria *	Berk.			Berkeley 1846
						* griseovinaceus *	A. Pearson ex Pegler			Pearson 1996
						* inandae *	Cooke			Cooke 1890
						* montagnei *	Kalchbr.	PREM	Yes	
						* nobilis *	(A. Pearson) Heinem.	PREM	Yes	
						* papilionaceus *	Bull.	PREM		
						* paurophyllus *	Berk.			Berkeley 1876
						* peroxydatus *	Berk.			Berkeley 1843
						* placomyces *	Peck	PREM	Yes	
						* pleropus *	Kalchbr. & MacOwan	PREM		
						* pogonatus *	Kalchbr.			Kalchbrenner 1881
						* purpurellus *	(F.H. Møller) F.H. Møller	PREM		Van der Westhuizen and Eicker 1988
						* sagittiformis *	Kalchbr. & Cooke			Kalchbrenner and Cooke 1881
						* semotus *	Fr.	PREM	Yes	
						* separatus *	L.	PREM		
						* silvaticus *	Schaeff.	PREM	Yes	
Fungi	Basidiomycota	Agaricomycetes	Agaricales	Agaricaceae	* Agaricus *	* silvicola *	(Vittad.) Peck		Yes	
						* sulphurellus *	Kalchbr. & Cooke	PREM		
						* trisulphuratus *	Berk.	PREM	Yes	
						* umbellifer * var. cinnabarinus	Berk.			Berkeley 1843
						* xanthodermus *	Genev.	PREM	Yes	
						* xanthodermus * var. griseus	(A. Pearson) Bon & Cappelli	PREM		
						* xanthodermus * var. lepiotoides	Maire		Yes	
						* xanthodermus * var. meleagrioides	(A. Pearson) Bon & Cappelli		Yes	
					* Arachnion *	* alborosella *	Verwoerd	PREM		
						* album *	Schwein.	PREM		
						* firmoderma *	Verwoerd	PREM		
						* scleroderma *	C.G. Loyd	PREM		
					* Battarrea *	* levispora *	Massee	PREM		
						* lycoperdon *	(Dicks.) Pers.	PREM		
						* phalloides *	(Dicks.) Pers.	PREM	Yes	As Battarrea stevenii
						* tepperiana *	F. Ludw.	PREM		
					* Battarreoides *	* diguetii *	(Pat. & Har.) R. Heim & T. Herrera	PREM		
					* Bovista *	* acocksii *	De Villiers, Eicker & Van der Westh.			De Villiers et al. 1989
						* capensis *	(Fr.) J.C. Coetzee & A.E. van Wyk			Coetzee & Van Wyk 2005
						* juglandiformis *	Berk. ex Massee			Massee 1888
						* lilacina *	Mont. & Berk.	PREM		
						* promontorii *	Kreisel			Kreisel 1967
						* umbrina *	Bottomley			Bottomley 1948
					* Calvatia *	* caelata *	(Bull.) Morgan	PREM		
						* candida *	(Rostk.) Hollós	PREM		
						* capensis *	(Lloyd) J.C. Coetzee, Eicker & A.E. van Wyk	PREM		
						* cyathiformis *	(Bosc) Morgan	PREM		
						* flava *	(Massee) Kreisel	PREM		
						* gigantea *	(Batsch) Lloyd	PREM		
						* incerta *	Bottomley			Bottomley 1948
						* lepidophora *	(Ellis & Everh.) Coker & Couch	PREM		
						* lilacina *	(Mont. & Berk.) Henn.	PREM	Yes	
						* rubroflava *	(Cragin) Lloyd	PREM		
					* Chlamydopus *	* meyenianus *	(Klotzsch) Lloyd	PREM		
					* Chlorophyllum *	* molybdites *	(G. Mey.) Massee	PREM	Yes	
Fungi	Basidiomycota	Agaricomycetes	Agaricales	Agaricaceae	* Chlorophyllum *	* africanum *	Z.W. Ge & A. Jacobs	PREM		Ge et al. 2018
						* palaeotropicum *	Z.W. Ge & A. Jacobs	PREM		Ge et al. 2018
					* Coniolepiota *	* spongodes *	(Berk. & Broome) Vellinga		Yes	
					* Coprinellus *	* curtus *	(Kalchbr.) Vilgalys, Hopple & Jacq. Johnson	PREM		
						* disseminatus *	(Pers.) J.E. Lange		Yes	
						* domesticus *	(Bolton) Vilgalys, Hopple & Jacq. Johnson		Yes	
						* ephemerus *	(Bull.) Redhead, Vilgalys & Moncalvo	PREM		
						* heptemerus *	(M. Lange & A.H. Sm.) Vilgalys, Hopple & Jacq. Johnson		Yes	
						* micaceus *	(Bull.) Vilgalys, Hopple & Jacq. Johnson	PREM	Yes	
						* truncorum *	(Scop.) Redhead, Vilgalys & Moncalvo	PREM		
					* Coprinopsis *	* atramentaria *	(Bull.) Redhead, Vilgalys & Moncalvo	PREM	Yes	
						* cinerea *	(Schaeff.) Redhead, Vilgalys & Moncalvo	PREM		
						* lagopus *	(Fr.) Redhead, Vilgalys & Moncalvo		Yes	
						* nivea *	(Pers.) Redhead, Vilgalys & Moncalvo	PREM	Yes	
						* picacea *	(Bull.) Redhead, Vilgalys & Moncalvo	PREM		Wood 2017
						* stercorea *	(Fr.) Redhead, Vilgalys & Moncalvo	PREM		
					* Coprinus *	* agricola *	A. Pearson			Pearson 1950
						* comatus *	(O.F. Müll.) Pers.	PREM	Yes	
						* digitalis *	(Batsch) Fr.	PREM		
						* papillatus *	(Batsch) Fr.	PREM		
						* punctatus *	Kalchbr.	PREM		
					* Crucibulum *	* vulgare *	Tul. & C. Tul.	PREM		
					* Disciseda *	* candida *	(Schwein.) Lloyd	PREM		
						* castanea *	(Lév.) Bottomley	PREM		
						* cervina *	(Berk.) G.H. Cunningham	PREM		
						* hypogaea *	(Cooke & Massee) G. Cunn.	PREM		
						* verrucosa *	G. Cunn.	PREM		
					* Gyrophragmium *	* delilei *	Mont	PREM		
					* Langermannia *	* wahlbergii *	(Fr.) Dring	PREM		
					* Lepiota *	* acutesquamosa *	(Weinm.) P. Kumm.	PREM		Van der Westhuizen and Eicker 1988
						* canescens *	A. Pearson	PREM		
						* citrinella *	Beeli	PREM		
					* Lepiota *	* cristata *	(Bolton) P. Kumm.	PREM	Yes	
						* cristatocystidiata *	A. Pearson	PREM		
Fungi	Basidiomycota	Agaricomycetes	Agaricales	Agaricaceae	* Lepiota *	* cutifracta *	A. Pearson	PREM		
						* flava *	Beeli	PREM		Van der Westhuizen and Eicker 1988
						* fustiformis *	A. Pearson	PREM		
						* goossensiae *	Beeli	PREM		Van der Westhuizen and Eicker 1988
						* helveola *	Bres.		Yes	
						* hispida *	Gillet	PREM		Van der Westhuizen and Eicker 1988
						* ianthina *	Sacc.	PREM		Van der Westhuizen and Eicker 1988
						* lutea *	Matt.	PREM		
						* morganii *	(Peck) Sacc.	PREM		
						* naucina * var. leucothites	(Vittad.) Sacc.	PREM		
						* nympharum *	(Kalchbr.) Kalchbr.	PREM		Van der Westhuizen and Eicker 1988
						* praeclara *	A. Pearson			Pearson 1950
						* parvannulata *	(Lasch) Gillet	PREM		Van der Westhuizen and Eicker 1988
						* rhizobola *	(Berk.) Sacc.	PREM		
						* roseolescens *	A. Pearson	PREM		
						* roseosquamosa *	Beeli	PREM		Van der Westhuizen and Eicker 1988
						* truncata *	A. Pearson	PREM		
						* umbrinozonata *	A. Pearson	PREM		
						* varians *	(Kalchbr. & MacOwan) Sacc.	PREM		
						* virescens *	Pat.	PREM		
					* Leucoagaricus *	* bisporus *	Heinem.	PREM	Yes	
						* leucothites *	(Vittad.) Wasser		Yes	
						* naucina *	(Vittad.) Wasser	PREM		Van der Westhuizen and Eicker 1988
						* rubrotinctus *	(Peck) Singer		Yes	
						* birnbaumii *	(Corda) Singer	PREM	Yes	
						* brebissonii *	(Godey) Locq.		Yes	
						* cepistipes *	(Sowerby) Pat.	PREM		
						* fragilissimus *	(Ravenel ex Berk. & M.A. Curtis) Pat.		Yes	
						* zeyheri *	(Berk.) Singer	PREM		
					* Lycoperdon *	* asperum *	(Lév.) Speg.	PREM		
						* caespitosum *	Welw. & Curr.	PREM		
						* caffrorum *	Kalchbr. & Cooke	PREM		
						* djurense *	Henn.	PREM		
						* duthiei *	Bottomley	PREM		
						* flavum *	Massee	PREM		
						* gunnii *	Berk.	PREM		
						* hiemale *	Vent.	PREM		
						* perlatum *	Pers.	PREM	Yes	
Fungi	Basidiomycota	Agaricomycetes	Agaricales	Agaricaceae	* Lycoperdon *	* polymorphum *	Vittad.	PREM		
						* pratense *	Pers.		Yes	As Vascellum pratense
						* pusillum *	Batsch	PREM		
						* qudenii *	Bottomley	PREM		
						* radicatum *	Durieu & Mont.	PREM		
						* subincarnatum *	Peck	PREM		
						* umbrinum *	Hornem.	PREM		
					* Macrolepiota *	* excoriatus *	(Schaeff.) Wasser	PREM		Van der Westhuizen and Eicker 1988
						* procera *	(Scop.) Singer	PREM	Yes	
						* prominens *	(Sacc.) M.M. Moser		Yes	
						* rhacodes *	(Vittad.) Singer		Yes	
						* zeyheri *	(Berk. & Singer) Heinem.	PREM	Yes	
					* Montagnea *	* aurenaria *	(DC.) Zeller			Reid and Eicker 1991
						* haussknechtii *	Rabenh.			Reid and Eicker 1991
					* Montagnites *	* candollei *	Speg.	PREM		
					* Mycenastrum *	* corium *	(Guers.) Desv.	PREM		
					* Parasola *	* hemerobia *	(Fr.) Redhead, Vilgalys & Hopple	PREM		
						* plicatilis *	(Curtis) Redhead, Vilgalys & Hopple	PREM	Yes	
					* Polyplocium *	* inquinans *	Berk.	PREM		
					* Psalliota *	* campestris *	(L.) Quél.	PREM		
						* alboargillascens *	A. Pearson	PREM		
						* arvensis *	Schaeff.	PREM		
						* arvensis * var. hortensis	W.G. Sm.	PREM		
						* comtula *	(Fr.) Quél.	PREM		
						* duriuscula *	Velen.	PREM		
						* mixta *	A. Pearson	PREM		
						* nobilis *	A. Pearson			Pearson 1950
						* placomyces *	Peck	PREM		
						* pratensis *	(Schaeff.) Gillet	PREM		
						* rodmanni *	(Peck) Kauffman	PREM		
						* volvata *	A. Pearson			Pearson 1950
						* xanthoderma * var. meleagrioides	A. Pearson	PREM		
					* Secotium *	* gueinzii *	Kunze			Kunze 1840
						* obtusum *	C.G. Loyd	PREM		
					* Tulostoma *	* albicans *	V.S. White		Yes	
						* bonianum *	Pat.		Yes	
						* cyclophorum *	Lloyd		Yes	
						* exasperatosporum *	J.E. Wright	PREM		
Fungi	Basidiomycota	Agaricomycetes	Agaricales	Agaricaceae	* Tulostoma *	* gracilipes *	J.E. Wright	PREM		
						* lesliei *	Van der Byl	PREM		
						* purpusii *	Henn.		Yes	
						* transvaalii *	C.G. Loyd	PREM		
					* Xanthagaricus *	* luteolosporus *	(Heinem. & Little Flower) Little Flower, Hosag. & T.K. Abraham	PREM	Yes	
				Amanitaceae	* Amanita *	* aureofloccosa *	Bas		Yes	
						* capensis *	Walleyn & Rammeloo		Yes	
						* excelsa *	(Fr.) Bertill.	PREM	Yes	
						* foetidissima *	D.A. Reid & Eicker	PREM	Yes	
						* mappa *	(Batsch) Bertill.		Yes	
						* muscaria *	(L.) Lam.	PREM	Yes	
						* pantherina *	(DC.) Krombh.	PREM	Yes	
						* phalloides *	(Vaill. ex Fr.) Link	PREM	Yes	
						* phalloides * var. alba	Costantin & L.M. Dufour		Yes	
						* phalloides * var. umbrina	(Ferry) Maire		Yes	
						* reidii *	Eicker & Greuning		Yes	
						* pleropus *	(Kalchbr. & MacOwan) D.A. Reid	PREM	Yes	
						* rubescens *	Pers.	PREM	Yes	
						* solitaria *	(Bull.) Fr.	PREM		
						* strobiliformis *	(Paulet ex Vittad.) Bertill.	PREM	Yes	
						* vaginata *	(Bull.) Lam.	PREM		
						* veldiei *	D.A. Reid & Eicker		Yes	
					* Limacella *	* guttata *	(Pers.) Konrad & Maubl.		Yes	
					* Saproamanita *	* praeclara *	(A. Pearson) Redhead, Vizzini, Drehmel & Contu	PREM	Yes	
				Bolbitiaceae	* Bolbitius *	* titubans *	(Bull.) Fr.	PREM	Yes	
						* vitellinus *	(Pers.) Fr.	PREM	Yes	
						* liberatus *	(Berk.) R. Heim	PREM		
					* Conocybe *	* apala *	(Fr.) Arnolds		Yes	
						* tenera *	(Schaeff.) Fayod	PREM	Yes	
					* Galeropsis *	* mitriformis *	(Berk.) R. Heim	PREM		
					* Pluteolus *	* reticulatus *	(Pers.) Gillet	PREM		Van der Westhuizen and Eicker 1988
				Broomeiaceae	* Broomeia *	* congregata *	Berk.	PREM	Yes	
						* ellipsospora *	Höhn.	PREM	Yes	
				Clavariaceae	* Clavaria *	* abietina *	Schumach.	PREM		
						* capensis *	Thunb.			Thunberg 1800
						* cinerea *	Bull.	PREM		
Fungi	Basidiomycota	Agaricomycetes	Agaricales	Clavariaceae	* Clavaria *	* cladoniae *	Kalchbr.	PREM		
						* contorta *	Holmsk.	PREM		
						* corniculata *	Schaeff.	PREM		
						* cristata *	(Holmsk.) Pers.	PREM		
						* flaccida *	Fr.	PREM		
						* helicoides *	Pat. & Demange	PREM		
						* kunzei *	Fr.	PREM		
						* laeticolor *	Berk. & M.A. Curtis	PREM		
						* ligula *	Schaeff.	PREM		
						* persimilis *	Cotton	PREM		
						* pulchra *	Peck	PREM		
						* stricta *	Schumach.	PREM		
					* Clavulinopsis *	* luteoalba *	(Rea) Corner		Yes	
						* ochracea *	Corner	PREM		
					* Mucronella *	* aggregata *	(Fr.) Fr.	PREM		
				Cortinariaceae	* Cortinarius *	* argutus *	Fr.	PREM		
						* brunneolimosus *	A. Pearson	PREM		
						* camurus *	Fr.	PREM		Van der Westhuizen and Eicker 1988
						* castaneus *	(Bull.) Fr.	PREM		
						* fuscotinctus *	Rea	PREM		Van der Westbuizen and Eicker 1988
						* lepidopus *	Cooke	PREM		Van der Westbuizen and Eicker 1988
						* multiformis *	(Fr.) Fr.	PREM		
						* radiofibrillosus *	A. Pearson	PREM		
					* Locellina *	* acetabulosa *	(Sowerby) Sacc.	PREM		
				Cyphellaceae	* Chondrostereum *	* purpureum *	(Pers.) Pouzar	PREM	Yes	
					* Cyphella *	* tabacina *	Cooke & W. Phillips	PREM		
						* applanata *	P.H.B. Talbot	PREM		
						* farinacea *	Kalchbr. & Cooke	PREM		
				Entolomataceae	* Claudopus *	* variabilis *	(Pers.) Fr.	PREM		
					* Clitopilus *	* prunulus *	(Scop.) P. Kumm.	PREM	Yes	
					* Entoloma *	* lividum *	(Bull.) Quél.	PREM		
						* olivipes *	A. Pearson ex Pegler			Pearson 1996
						* sagittiforme *	(Kalchbr. & Cooke) Sacc.	PREM		
				Fistulinaceae	* Fistulina *	* africana *	Van der Byl	PREM	Yes	
				Hydnangiaceae	* Laccaria *	* amethystea *	(Bull.) Murrill	PREM	Yes	
						* laccata *	(Scop.) Cooke	PREM	Yes	
						* tortilis *	(Bolton) Cooke		Yes	
				Hygrophoraceae	* Hygrocybe *	* aurantiorufa *	A. Pearson ex Pegler			Pearson 1996
						* chlorophana *	(Fr.) Wünsche		Yes	
Fungi	Basidiomycota	Agaricomycetes	Agaricales	Hygrophoraceae	* Hygrocybe *	* conica *	Velen.	PREM	Yes	
						* nigrescens *	(Quél.) Kühner	PREM	Yes	
						* zuluensis *	Boertm.			Boertman 1998
					* Hygrophorus *	* coccineus *	(Schaeff.) Fr.	PREM		
						* conicus *	(Schaeff.) Fr.	PREM		
						* conicus * var. nigrescens	(Quél.) Konrad & Maubl.	PREM		
							(Quél.) Konrad & Maubl.	PREM		
						* discolor *	(Feltgen) Sacc. & Trotter	PREM		
				Incertae sedis	* Anellaria *	* separata *	(L.) P. Karst.	PREM		
					* Panaeolina *	* foenisecii *	(Pers.) Maire	PREM	Yes	
					* Panaeolus *	* caliginosus *	(Jungh.) Gillet	PREM		
						* campanulatus *	(L.) Quél.	PREM		Van der Westhuizen and Eicker 1988
						* fimicola *	(Pers.) Gillet	PREM		
						* fimicoloides *	A. Pearson	PREM		
						* papilionaceus *	(Bull.) Quél.	PREM	Yes	
						* retirugus *	(Fr.) Gillet	PREM		
						* semiovatus *	(Sowerby) S. Lundell & Nannf.		Yes	
						* semiovatus * f. exannulatus	A. Pearson			Pearson 1950
						* solidipes *	(Peck) Sacc.	PREM		
						* sphinctrinus *	(Fr.) Quél.	PREM		
						* subbalteatus *	(Berk. & Broome) Sacc.		Yes	
				Inocybaceae	* Astrosporina *	* maritima *	(P. Karst.) Rea	PREM		Van der Westhuizen and Eicker 1988
					* Crepidotus *	* austroafricanus *	Pilát			Pilát 1950
						* haustellaris *	(Fr.) P. Kumm.	PREM		
						* inandae *	Cooke	PREM		
						* mollis *	(Schaeff.) Staude	PREM	Yes	
						* pogonatus *	Kalchbr.	PREM		
						* variabillis *	(Pers.) P. Kumm.	PREM	Yes	
					* Inocybe *	* cinnamomea *	A. Pearson ex Pegler			Pearson 1996
						* congregata *	A. Pearson			Pearson 1950
						* curvipes *	P. Karst.			Vellinga et al. 2009
				Inocybaceae	* Inocybe *	* eutheles *	Sacc.	PREM	Yes	
						* hirtella *	Bres.		Yes	
						* lanuginella *	(J. Schröt.) Konrad & Maubl		Yes	
						* microspora *	J.E. Lange	PREM		Van der Westhuizen and Eicker 1988
						* mixtilis *	(Britzelm.) Sacc.	PREM		Van der Westhuizen and Eicker 1988
						*	Gillet		Yes	
						* patouillardii *	Bres.		Yes	
Fungi	Basidiomycota	Agaricomycetes	Agaricales	Inocybaceae	* Inocybe *	* pullata *	A. Pearson ex Pegler			Pearson and Pegler 1996
					* Phaeoglabrotricha *	* farinacea *	(Kalchbr. & Cooke) W.B. Cooke	PREM		
					* Phaeosolenia *	* densa *	(Berk.) W.B. Cooke	PREM		
				Lyophyllaceae	* Lyophyllum *	* decastes *	(Fr.) Singer		Yes	
					* Podabrella *	* microcarpa *	(Berk. & Broome) Singer	PREM		
					* Termitomyces *	* clypeatus *	R. Heim		Yes	
						* microcarpus *	(Berk. & Broome) R. Heim		Yes	
						* reticulatus *	Van der Westh. & Eicker		Yes	
						* sagittiformis *	(Kalchbr. & Cooke) D.A. Reid		Yes	
						* schimperi *	(Pat.) R. Heim		Yes	
						* umkowaan *	(Cooke & Massee) D.A. Reid		Yes	
				Marasmiaceae	* Calyptella *	* capensis *	W.B. Cooke & P.H.B. Talbot	PREM		
						* capensis *	(Berk.) D.A. Reid		Yes	
					* Marasmius *	* bekolacongoli *	Beeli		Yes	
						* calopus *	(Pers.) Fr.	PREM		
						* candidus *	(Bolton) Fr.	PREM		
						* delectans *	Morgan	PREM		Van der Westhuizen and Eicker 1988
						* epiphyllus *	(Pers.) Fr.	PREM		Van der Westhuizen and Eicker 1988
						* filaris *	Kalchbr. & MacOwan	PREM		
						* haematocephalus *	(Mont.) Fr.		Yes	
						* helvolus *	Berk.	PREM		
						* oreades *	(Bolton) Fr.		Yes	
						* oreadoides *	Pass.	PREM		
						* petalinus *	Berk. & M.A. Curtis	PREM		
						* rotula *	(Scop.) Fr.	PREM		Van der Westhuizen and Eicker 1988
						* scorodonius *	(Fr.) Fr.	PREM		Van der Westhuizen and Eicker 1988
						* siccus *	(Schwein.) Fr.	PREM		Van der Westhuizen and Eicker 1988
						* tener *	Berk. & M.A. Curtis	PREM		
						* thwaitesii *	Berk. & Broome	PREM		
						* titanosporus *	D.A. Reid & Jacot Guill.			Reid and Guillarmod 1988
				Marasmiaceae	* Marasmius *	* zenkeri *	Henn.	PREM		
					* Solenia *	* minima *	Cooke & W. Phillips	PREM		
						* natalensis *	W.B. Cooke	PREM		
						* rhoina *	W.B. Cooke	PREM		
				Mycenaceae	* Cruentomycena *	* viscidocruenta *	(Cleland) R.H. Petersen & Kovalenko		Yes	
					* Favolaschia *	* thwaitesii *	(Berk. & Broome) Kuntze	PREM	Yes	
					* Mycena *	* acicula *	(Schaeff.) P. Kumm.	PREM		Van der Westhuizen and Eicker 1988
						* aetites *	(Fr.) Quél.		Yes	
Fungi	Basidiomycota	Agaricomycetes	Agaricales	Mycenaceae	* Mycena *	* alcalina *	(Fr.) P. Kumm.	PREM		
						* alcalinoides *	A. Pearson	PREM		
						* clavicularis *	(Fr.) Gillet	PREM		
						* corticola *	(Schumach.) Quél.	PREM		Van der Westhuizen and Eicker 1988
						* hiemalis *	(Osbeck) Quél.	PREM		
						* hyalina *	A. Pearson	PREM		
						* pura *	(Pers.) P. Kumm.		Yes	
						* rhodiophylla *	(Kalchbr.) Sacc.	PREM		
						* rubromarginata *	(Fr.) P. Kumm.	PREM		
						* sciola *	(Kalchbr.) Sacc.	PREM		
						* vibecina *	A. Pearson	PREM	Yes	
				Niaceae	* Flagelloscypha *	* applanata *	(P.H.B. Talbot) W.B. Cooke	PREM		
					* Lachnella *	* alboviolascens *	(Alb. & Schwein.) Fr.	PREM		
				Nidulariaceae	* Cyathus *	* berkeleyanus *	(Tul. & C. Tul.) Lloyd	PREM		
						* dasypus *	Nees	PREM		
						* hookeri *	Berk.	PREM		
						* microsporus *	Tul. & C. Tul.	PREM		
						* minutosporus *	Lloyd	PREM		
						* montagnei *	Tul. & C. Tul.	PREM		
						* olla *	(Batsch) Pers.	PREM	Yes	
						* pallidus *	Berk. & M.A. Curtis	PREM		
						* poeppigii *	Tul. & C. Tul.	PREM		
						* stercoreus *	(Schwein.) De Toni	PREM		
						* striatus *	(Huds.) Willd.		Yes	
						* vernicosus *	(Bull.) DC.	PREM		
				Omphalotaceae	* Anthracophyllum *	* nigritum *	(Lév.) Kalchbr.	PREM		
						* archeri *	(Berk.) Pegler		Yes	
					* Gymnopus *	* androsaceus *	(L.) Della Magg. & Trassin.	PREM	Yes	
					* Marasmiellus *	* candidus *	(Fr.) Singer		Yes	
					* Omphalotus *	* olearius *	(DC.) Singer		Yes	
				Phelloriniaceae	* Phellorinia *	* inquinans *	Berk.	PREM		
						* squamosa *	Kalchbr. & MacOwan	PREM		
						* strobilina *	(Kalchbr.) Kalchbr.	PREM		
				Physalacriaceae	* Armillaria *	* fuscipes *	Petch.		Yes	Coetzee et al. 2000
						* gallica *	Marxm.			Coetzee et al. 2003
						* mellea *	(Vahl) P. Kumm.	PREM	Yes	Coetzee et al. 2000
						* ramentacea *	(Bull.) Gillet	PREM		
					* Armillariella *	* polymyces *	(Pers.) Singer & Clémençon		Yes	
					* Cyptotrama *	* asprata *	(Berk.) Redhead & Ginns	PREM	Yes	
Fungi	Basidiomycota	Agaricomycetes	Agaricales	Physalacriaceae	* Oudemansiella *	* canarii *	(Jungh.) Höhn.	PREM		
					* Hymenopellis *	* radicata *	(Relhan) Singer	PREM		Van der Westhuizen and Eicker 1988
					* Physalacria *	* decaryi *	Pat.	PREM		
					* Xerula *	* atrocaerulea *	R.H. Petersen & Bougher			Petersen and Bougher 2008
				Pleurotaceae	* Pleurotus *	* applicatus *	(Batsch) P. Kumm.	PREM		
						* geesterani *	Singer			Singer 1962
						* gilvescens *	(Kalchbr.) Sacc.	PREM		
						* lenticula *	(Kalchbr.) Sacc.	PREM		
						* limpidus *	(Fr.) Sacc.	PREM		
						* ostreatus *	(Jacq.) P. Kumm.	PREM	Yes	
						* perpusillus *	(Lumn.) Gillet	PREM		
						* pulmonarius *	(Fr.) Quél.		Yes	
						* sajor-caju *	(Fr.) Singer	PREM		
						* sciadium *	(Kalchbr. & MacOwan) Sacc.	PREM		
						* septicus *	(Fr.) P. Kumm.	PREM		
				Pluteaceae	* Pluteus *	* atromarginatus *	(Konrad) Kühner	PREM		
						* pellitus *	(Pers.) P. Kumm.	PREM		
						* romelli *	(Britzelm.) Sacc.		Yes	
						* salicinus *	(Pers.) P. Kumm.		Yes	
						* semibulbosus *	(Lasch) Gillet		Yes	
						* thomsonii *	(Berk. & Broome) Dennis	PREM		
					* Volvariella *	* speciosa *	(Fr.) Singer		Yes	
				Podaxaceae	* Podaxis *	* africana *	De Villiers, Eicker & Van der Westh.	PREM		
						* pistillaris *	(L.) Fr.	PREM	Yes	
						* rugospora *	De Villiers, Eicker & Van der Westh.	PREM		
				Psathyrellaceae	* Ozonium *	* omnivorum *	Shear	PREM		
					* Psathyrella *	* griseola *	A. Pearson	PREM		
						* condolleana *	(Fr.) Maire		Yes	
						* lionella *	A. Pearson ex Pegler			Pearson 1996
				Psathyrellaceae	* Psathyrella *	* praelonga *	A. Pearson			Pearson 1950
						* varicosa *	A. Pearson			Pearson 1950
				Pterullaceae	* Pterula *	* subulata *	Fr.		Yes	
				Schizophillaceae	* Schizophyllum *	* commune *	Fr.		Yes	
				Sebacinaceae	* Sebacina *	* schweinitzii *	(Peck) Oberw.		Yes	
				Strophariaceae	* Agrocybe *	* praecox *	(Pers.) Fayod	PREM	Yes	
						* pediades *	(Fr.) Fayod		Yes	
					* Deconica *	* atrorufa *	(Schaeff.) P. Karst.	PREM		
						* coprophila *	(Bull.) Fr.	PREM		
Fungi	Basidiomycota	Agaricomycetes	Agaricales	Strophariaceae	* Deconica *	* protea *	(Kalchbr.) Desjardin & B.A. Perry			Kalchbrenner 1876 (as *Agaricus proteus*)
					* Flammula *	* alnicola *	(Fr.) P. Kumm.	PREM		
						* harmoge *	(Fr.) Sacc.	PREM		
						* hybrida *	(Bull.) Gillet	PREM		
						* laetilamellata *	A. Pearson	PREM		
						* luxurians *	A. Pearson	PREM		
						* papillata *	A. Pearson	PREM		
						* penetrans *	(Fr.) Quél.	PREM		
						* sapinea *	(Fr.) Pat.	PREM		
					* Galera *	* hypnorum *	(Batsch) Quél.	PREM		
						* lateritia *	(Fr.) P. Kumm.	PREM		
						* pygmaeoaffinis *	(Fr.) Quél.	PREM		Van der Westhuizen and Eicker 1988
						* spartea *	Velen.	PREM		Van der Westhuizen and Eicker 1988
						* tenera *	(Schaeff.) P. Kumm.	PREM		
						* tenera * var. siliginea	(Fr.) P. Kumm.	PREM		
					* Gymnopilus *	* hybridus *	(Bull.) Maire		Yes	
						* junonius *	(Fr.) P.D. Orton	PREM	Yes	
						* penetrans *	(Fr.) Murrill	PREM	Yes	
						* sapineus *	(Fr.) Murrill		Yes	
					* Hebeloma *	* angustispermum *	A. Pearson	PREM		
						* anthracophilum *	Maire	PREM		
						* crustuliniforme *	(Bull.) Quél.		Yes	
						* cylindrosporum *	Romagn.	PREM	Yes	
						* nudipes *	(Fr.) Kalchbr.	PREM		
						* sinapizans *	(Paulet) Gillet		Yes	
						* spoliatum *	(Fr.) Gillet	PREM		
					* Hymenogaster *	* albellus *	Massee & Rodway	PREM		
						* reticulatus *	Zeller & C.W.	PREM		
					* Hypholoma *	* candolleanum *	(Fr.) Quél.	PREM		
						* fasciculare *	(Huds.) P. Kumm.	PREM	Yes	
						* lateritium *	(Schaeff.) P. Kumm.	PREM		
					* Kuehneromyces *	* mutabilis *	(Schaeff.) Singer & A.H. Sm.	PREM		
					* Leratiomyces *	* ceres *	(Cooke & Massee) Spooner & Bridge 2008		Yes	
					* Naucoria *	* pediades *	(Fr.) P. Kumm.	PREM		
						* russa *	(Cooke & Massee) Sacc.	PREM		
						* scolecina *	(Fr.) Quél.	PREM		
						* semiorbicularis *	(Bull.) Quél.	PREM		
Fungi	Basidiomycota	Agaricomycetes	Agaricales	Strophariaceae	* Naucoria *	* undulosa *	(Fr.) Sacc.	PREM		
					* Pholiota *	* aurivella *	(Batsch) P. Kumm.	PREM		
						* caperata *	(Pers.) Gillet	PREM		
						* cylindracea *	(DC.) Gillet	PREM		
						* flammans *	(Batsch) P. Kumm.	PREM		
						* mutabilis *	(Schaeff.) P. Kumm.	PREM		
						* parva *	A. Pearson	PREM		
						* pseudoerebia *	A. Pearson	PREM		
						* squarrosa *	(Oeder) P. Kumm.		Yes	
						* spectabilis *	(Fr.) P. Kumm.	PREM		
						* togularis *	(Bull.) P. Kumm.	PREM		
						* unicolor *	(Vahl) Gillet	PREM		
					* Psilocybe *	* coprophila *	(Bull.) P. Kumm.		Yes	
						* cylindrispora *	A. Pearson			Pearson 1950
						* natalensis *	Gartz, D.A. Reid, M.T. Sm. & Eicker	PREM		
					* Stropharia *	* coccinea *	A. Pearson ex Pegler			Pearson 1996
						* semiglobata *	(Batsch) Quél.		Yes	
					* Tubaria *	* furfuracea *	(Pers.) Gillet		Yes	
				Tricholomataceae	* Amparoina *	* spinosissima *	(Singer) Singer		Yes	
					* Cellypha *	* rhoina *	(W.B. Cooke) W.B. Cooke	PREM		
					* Clitocybe *	* expallens *	(Pers.) P. Kumm.	PREM		
						* fragrans *	(With.) P. Kumm.	PREM		
						* gentianea *	Quél.	PREM		
						* nuda *	(Bull.) H.E. Bigelow & A.H. Sm.	PREM		Van der Westhuizen and Eicker 1988 (as *Lepista nuda*)
						* rivulosa *	(Pers.) P. Kumm.	PREM		
						* splendens *	(Pers.) Gillet	PREM		
						* toxica *	Stephens			Stephens 1966
					* Collybia *	* acervata *	(Fr.) P. Kumm.	PREM		
						* albuminosa *	(Berk.) Petch	PREM		Van der Westhuizen and Eicker 1988
						* butyracea *	(Bull.) P. Kumm.	PREM		Van der Westhuizen and Eicker 1988
						* chrysopepla *	(Berk. & M.A. Curtis) A. Pearson	PREM		
						* distorta *	(Fr.) Quél.	PREM	Yes	
						* dryophila *	(Bull.) P. Kumm.	PREM	Yes	
						* extuberans *	(Fr.) Quél.	PREM		
						* extuberans *	(Fr.) Quél.	PREM		
						* fragrantissima *	A. Pearson	PREM		
						* fusipes *	(Bull.) Quél.	PREM	Yes	
						* macilenta *	(Fr.) Gillet	PREM		
Fungi	Basidiomycota	Agaricomycetes	Agaricales	Tricholomataceae	* Collybia *	* maculatoides *	A. Pearson	PREM		
						* ocellata *	(Fr.) P. Kumm.	PREM		
						* radicata *	(Relhan) Quél.	PREM		
						* stridula *	(Fr.) Sacc.	PREM		
						* velutipes *	(Curtis) P. Kumm.	PREM		
					* Lepista *	* caffrorum *	(Kalchbr. & MacOwan) Singer	PREM	Yes	
						* sordida *	(Schumach.) Singer	PREM	Yes	
					* Macrocybe *	* lobayensis *	(R. Heim) Pegler & Lodge	PREM	Yes	
						* titans *	(H.E. Bigelow & Kimbr.) Pegler, Lodge & Nakasone	PREM		
					* Melanoleuca *	* brevipes *	(Bull.) Pat.	PREM		
						* melaleuca *	(Pers.) Murrill	PREM		
					* Omphalia *	* glaucophylla *	(Lasch) Sacc.	PREM		Van der Westhuizen and Eicker 1988
						* micromeles *	(Berk. & Broome) Sacc.	PREM		
						* oniscus *	(Fr.) Gillet	PREM		
						* pyxidatoides *	A. Pearson	PREM		
						* rustica *	(Fr.) Quél.	PREM		
					* Tricholoma *	* albobrunneum *	(Pers.) P. Kumm.		Yes	
						* eucalypticum *	A. Pearson			Pearson 1950
						* melaleucum * f. acystidiatum	A. Pearson	PREM		
						* meridianum *	A. Pearson			Pearson 1950
						* saponaceum *	(Fr.) P. Kumm.		Yes	
						* ustale *	(Fr.) P. Kumm.		Yes	
					* Tricholomopsis *	* rutilans *	(Schaeff.) Singer		Yes	
					* Tricholosporum *	* laeteviolaceum *	D.A. Reid, Eicker, Clémençon & Cec. Roux	PREM		
					* Trogia *	* cantharelloides *	(Mont.) Pat.		Yes	
			Auriculariales	Auriculariaceae	* Auricularia *	* auricula-judae *	(Bull.) Quél.	PREM	Yes	
						* delicata *	(Mont.) Henn.	PREM		
						* emini *	Henn.	PREM		
						* fuscosuccinea *	(Mont.) Henn.	PREM		
						* mesenterica *	(Dicks.) Pers.	PREM		
						* polytricha *	(Mont.) Sacc.	PREM		
						* sambucina *	Mart.	PREM		
					* Eichleriella *	* macrospora *	(Ellis & Everh.) G.W. Martin	PREM		
					* Exidia *	* glandulosa *	(Bull.) Fr.	PREM	Yes	
						* purpureocinerea *	MacOwan & Kalchbr.	PREM		
					* Heterochaete *	* byliana *	Talbot	PREM		
Fungi	Basidiomycota	Agaricomycetes	Auriculariales	Auriculariaceae	* Heterochaete *	* grandispora *	P.H.B. Talbot	PREM		
				Incertae sedis	* Aporpium *	* caryae *	(Schwein.) Teixeira & D.P. Rogers	PREM		
			Boletales	Boletaceae	* Aureoboletus *	* gentilis *	(Earle) Klofac		Yes	
					* Boletus *	* aureus *	Schaeff.		Yes	
						* aestivalis *	(Paulet) Fr.	PREM	Yes	
						* bovinus *	Rostk.	PREM		
						* bovinus * var. viridocaerulescens	A. Pearson	PREM		
						* collinitus *	Fr.	PREM		
						* edulis *	Rostk.	PREM	Yes	
						* flavus *	Pollini	PREM		
						* grevillei *	Klotzsch	PREM		
						* curtipes *	Massee			Massee 1908
						* pinicola *	Sw.		Yes	
						* reticulatus *	Schaeff.		Yes	
						* stellenbossiensis *	Van der Byl			Van der Byl 1925
						* subflammeus *	Berk.			Berkeley 1876
					* Buchwaldoboletus *	* hemichrysus *	(Berk. & M.A. Curtis) Singer		Yes	
					* Chalciporus *	* piperatus *	(Bull.) Bataille	PREM	Yes	
					* Imleria *	* badia *	(Fr.) Vizzini	PREM	Yes	
					* Leccinum *	* duriusculum *	(Schulzer ex Kalchbr.) Singer	PREM	Yes	
					* Octaviania *	* africana *	Lloyd	PREM		
						* flava *	(Rodway) G. Cunn.	PREM		
					* Xerocomellus *	* chrysenteron *	(Bull.) Šutara		Yes	
				Boletinellaceae	* Phlebopus *	* sudanicus *	(Har. & Pat.) Heinem.	PREM	Yes	
				Boletinellaceae	* Phlebopus *	* colossus *	(R. Heim) Singer		Yes	
				Coniophoraceae	* Coniophora *	* arida *	(Fr.) P. Karst.	PREM		
						* cerebella *	(Pers.) Pers.	PREM		
						* fodinarum *	P.H.B. Talbot	PREM		
						* incrustata *	P.H.B. Talbot	PREM		
						* mollis *	Ginns			Ginns 1982
						* olivacea *	Massee	PREM		
						* papillosa *	P.H.B. Talbot	PREM		
					* Gyrodontium *	* capense *	D.A. Reid			Reid 1963
				Gyroporaceae	* Gyroporus *	* castaneus *	(Bull.) Quél.	PREM	Yes	
				Paxillaceae	* Melanogaster *	* ambiguus *	(Vittad.) Tul. & C. Tul.	PREM		
					* Paxillus *	* extenuatus *	Fr.	PREM		
						* involutus *	(Batsch) Fr.	PREM	Yes	
Fungi	Basidiomycota	Agaricomycetes	Boletales	Paxillaceae	* Paxillus *	* panuoides *	(Fr.) Fr.	PREM	Yes	
				Rhizopogonaceae	* Rhizopogon *	* capensis *	C.G. Loyd	PREM		
						* luteolus *	Fr. & Nordholm		Yes	
				Sclerodermataceae	* Pisolithus *	* tinctorius *	(Mont.) E. Fisch.	PREM	Yes	
					* Scleroderma *	* capense *	C.G. Loyd	PREM		
						* cepa *	Pers.		Yes	
						* citrinum *	Pers.		Yes	
						* flavidum *	Ellis & Everh.		Yes	
						* verrucosum *	(Bull.) Pers.		Yes	
						* stellenbosiensis *	Verwoerd	PREM		
				Serpulaceae	* Serpula *	* himantioides *	(Fr.) P. Karst.		Yes	
				Suillaceae	* Suillus *	* bellinii *	(Inzenga) Watling		Yes	
						* bovinus *	(L.) Roussel		Yes	
						* granulatus *	(L.) Roussel		Yes	
						* luteus *	(L.) Roussel		Yes	
			Cantharellales	Cantharellaceae	* Cantharellus *	* cibarius *	Fr.	PREM		
						* longisporus *	Heinem.		Yes	
				Ceratobasidiaceae	* Pellicularia *	* asperula *	D.P. Rogers	PREM		
						* filamentosa *	(Pat.) D.P. Rogers	PREM		
						* fodinarum *	P.H.B. Talbot & V.C. Green	PREM		
						* vaga *	(Berk. & M.A. Curtis) D.P. Rogers ex Linder	PREM		
				Clavulinaceae	* Clavulina *	* cinerea *	(Bull.) J. Schröt.	PREM		
						* cristata *	(Holmsk.) J. Schröt.	PREM	Yes	
			Corticiales	Corticiaceae	* Corticium *	* argillaceum *	Bres.	PREM		
					* Corticium *	* armeniacum *	Sacc.	PREM		
						* coeruleum *	(Lam.) Fr.	PREM		
						* confluens *	(Fr.) Fr.	PREM		
						* gloeosporum *	P.H.B. Talbot	PREM		
						* laetum *	(P. Karst.) Bres.	PREM		
						* luteocystidiatum *	P.H.B. Talbot	PREM		
						* moniliforme *	P.H.B. Talbot	PREM		
						* portentosum *	Berk. & M.A. Curtis	PREM		
						* punctulatum *	Cooke	PREM		
						* salmonicolor *	Berk. & Broome	PREM		
						* scutellare *	Berk. & M.A. Curtis	PREM		
						* tumulosum *	P.H.B. Talbot	PREM		
						* vagum *	Berk. & M.A. Curtis	PREM		
					* Cytidia *	* flocculenta *	(Fr.) Höhn. & Litsch.	PREM		
Fungi	Basidiomycota	Agaricomycetes	Corticiales	Corticiaceae	* Dendrothele *	* duthiei *	P.H.B. Talbot	PREM		
					* Laetiporus *	* baudonii *	(Pat.) Ryvarden		Yes	
						* sulphureus *	(Bull.) Murrill	PREM	Yes	
					* Tretopileus *	* sphaerophorus *	(Berk. & M.A. Curtis) S. Hughes & Deighton	PREM		
			Geastrales	Geastraceae	* Geasteropsis *	* conrathi *	Hollós	PREM		
					* Geastrum *	* ambiguum *	Mont.	PREM		
						* arenarium *	Lloyd	PREM		
						* bryantii *	Berk.	PREM		
						* campestre *	Morgan	PREM		
						* coronatum *	Pers.	PREM		
						* dissimile *	Bottomley	PREM		
						* fimbriatum *	Tul. & C. Tul.	PREM		
						* floriforme *	Vittad.	PREM		
						* fornicatum *	(Huds.) Hook.	PREM		
						* hieronymi *	Henn.	PREM		
						* hygrometricum *	Pers.	PREM		
						* kotlabae *	V.J. Staněk		Yes	
						* lageniforme *	Cooke	PREM		
						* limbatum *	Fr.	PREM	Yes	
						* mammosum *	De Toni	PREM		
						* minimum *	Chevall.	PREM		
						* mirabile *	Mont.	PREM		
						* nanum *	Pers.	PREM		
						* pectinatum *	Pers.	PREM	Yes	
						* pouzarii *	V.J. Staněk	PREM		
						* quadrifidum *	DC. ex Pers.	PREM		
						* rabenhorstii *	Kunze	PREM		
						* saccatum *	Speg.	PREM	Yes	
						* schmidelii *	Vittad.	PREM		
						* schweinitzii *	(Berk. & M.A. Curtis) Zeller		Yes	
						* sessile *	(Sowerby) Pouzar		Yes	
						* smardae *	V.J. Staněk	PREM		
						* striatum *	Quél.	PREM		
						* triplex *	Jungh.	PREM	Yes	
						* velutinum *	Morgan	PREM		
					* Myriostoma *	* coliforme *	(Dicks.) Corda	PREM	Yes	
						* coliforme * var. capillisporum	V.J. Staněk			Staněk 1958
Fungi	Basidiomycota	Agaricomycetes	Gloeophyllales	Gloeophyllaceae	* Gloeophyllum *	* sepiarium *	(Wulfen) P. Karst.	PREM	Yes	
						* trabeum *	(Pers.) Murrill	PREM	Yes	
			Gomphales	Gomphaceae	* Ramaria *	* formosa *	(Pers.) Quél.		Yes	
				Clavariadelphaceae	* Clavariadelphus *	* clavulinoides *	R.H. Petersen			Petersen 1967
			Hymenochaetales	Hymenochaetaceae	* Coltricia *	* perennis *	(L.) Murrill	PREM	Yes	
					* Fomitoparia *	* capensis *	M. Fisch., Cloete, L. Mostert & Halleen			Cloete et al. 2014
					* Fomitoparia *	* punctata *	(P. Karst.) Murrill		Yes	
					* Fuscoporia *	* gilva *	(Schwein.) T. Wagner & M. Fisch.		Yes	
					* Hydnum *	* auriscalpium *	Lour.	PREM		
						* longospinosum *	Lloyd	PREM		
						* mucidum *	Pers.	PREM		
						* sclerodontium *	Mont. & Berk.	PREM		
						* setosum *	Pers.	PREM		
					* Hymenochaete *	* cinnamomea *	(Pers.) Bres.	PREM		
						* contiformis *	G. Cunn.	PREM		
						* fasciculata *	P.H.B. Talbot	PREM		
						* fulva *	Burt	PREM		
						* luteobadia *	(Fr.) Höhn. & Litsch.	PREM		
						* ochromarginata *	P.H.B. Talbot	PREM	Yes	
						* pinnatifida *	Burt	PREM		
						* rubiginosa *	(Dicks.) Lév.	PREM	Yes	
						* semistupposa *	Petch	PREM		
						* tabacina *	(Sowerby) Lév.	PREM		
			Hymenochaetales	Hymenochaetaceae	* Hymenochaete *	* tristicula *	(Berk. & Broome) Massee	PREM		
					* Phellinus *	* gilvus *	(Schwein.) Pat.	PREM	Yes	
						* igniarius *	(L.) Quél.		Yes	
						* resupinatus *	M. Fisch., Cloete, L. Mostert & Halleen			Cloete et al. 2016
						* rimosus *	(Berk.) Pilát	PREM	Yes	
					* Polystictus *	* albobadius *	C.G. Loyd	PREM		
						* doidgei *	C.G. Loyd	PREM		
						* subiculoides *	C.G. Loyd	PREM		
					* Trichaptum *	* byssogenum *	(Jungh.) Ryvarden		Yes	
			Incertae sedis	Incertae sedis	* Cotylidia *	* aurantiaca * f. infundibuliformis	D.A. Reid	PREM		
					* Grandinia *	* bicolor *	P.H.B. Talbot	PREM		
					* Heterochaete *	* byliana *	P.H.B. Talbot	PREM		
					* Heterochaete *	* grandispora *	P.H.B. Talbot	PREM		
					* Oxyporus *	* populinus *	(Schumach.) Donk	PREM		
Fungi	Basidiomycota	Agaricomycetes	Incertae sedis	Incertae sedis	* Riessia *	* semiophora *	Fresen.	PREM		
			Phallales	Phallaceae	* Anthurus *	* archeri *	(Berk.) E. Fisch.	PREM		
					* Aseroë *	* rubra *	Labill.	PREM	Yes	
					* Blumenavia *	* angolensis *	(Welw. & Curr.) Dring		Yes	
					* Clathrella *	* roseolescens *	E. Fisch.	PREM		
					* Clathrus *	* affinis *	Lloyd	PREM		
						* archeri *	(Berk.) Dring	PREM	Yes	
						* gracilis *	(Berk.) Schltdl.	PREM		
						* pseudocancellatus *	(E. Fisch.) Lloyd	PREM		
						* transvaalensis *	Eicker & D.A. Reid		Yes	
					* Ileodictyon *	* gracile *	Berk.			Bottomley 1948; Coetzee 2010
					* Itajahya *	* galericulata *	Möller	PREM	Yes	
						* rosea *	(Delile) E. Fisch.		Yes	
					* Jaczewskia *	* phalloides *	Mattir.	PREM		
					* Kalchbrennera *	* corallocephala *	(Welw. & Curr.) Kalchbr.	PREM	Yes	
					* Lysurus *	* cruciatus *	(Lepr. & Mont.) Henn.			Bottomley 1948; Coetzee 2010
						* gardneri *	Berk.	PREM		
					* Mutinus *	* bambusinus *	(Zoll.) E. Fisch.	PREM		
						* caninus *	(Huds.) Fr.	PREM		
						* simplex *	Lloyd.	PREM		
					* Phallus *	* duplicatus *	Bosc		Yes	
						* impudicus *	L.	PREM	Yes	
						* indusiatus *	Vent.	PREM	Yes	
					* Phallus *	* rubicundus *	(Bosc) Fr.	PREM	Yes	
			Polyporales	Fomitopsidaceae	* Daedalea *	* biennis *	(Bull.) Fr.	PREM		
						* hobbsii *	Van der Byl	PREM		
						* quercina *	(L.) Pers.	PREM	Yes	
					* Fomitopsis *	* ochroleuca *	(Berk.) G. Cunn.	PREM		
					* Gloeocystidium *	* tenue *	(Pat.) Höhn. & Litsch.	PREM		
					* Phaeolus *	* schweinitzii *	(Fr.) Pat.	PREM	Yes	
					* Rhodofomitopsis *	* lilacinogilva *	(Berk.) B.K. Cui, M.L. Han & Y.C. Dai	PREM	Yes	
				Ganodermataceae	* Amauroderma *	* leptopus *	(Pers.) J.S. Furtado	PREM		
						* fuscoporia *	Wakef.			Wakefield 1948
						* rude *	(Berk.) Torrend	PREM	Yes	
						* schomburgkii *	(Mont. & Berk.) Torrend	PREM		
						* sprucei *	(Pat.) Torrend		Yes	
						* zuluense *	Talbot	PREM		
					* Ganoderma *	* alluaudi *	Pat. & Har.	PREM		
						* annulare *	(Fr.) Gilb.	PREM		
Fungi	Basidiomycota	Agaricomycetes	Polyporales	Ganodermataceae	* Ganoderma *	* applanatum *	(Pers.) Pat.	PREM	Yes	
						* aridicola *	J.H. Xing & B.K. Cui			Xing et al. 2016
						* austroafricanum *	Coetzee, M.J. Wingf., Marinc., Blanchette			Crous et al. 2014
						* chilense *	(Fr.) Pat.	PREM		
						* colossus *	Fr.) C.F. Baker	PREM		
						*cf. cupreum*	(Sacc.) Bres.			Tchotet et al. 2019
						* curtisii *	(Berk.) Murrill	PREM		
						* destructans *	M.P.A. Coetzee, Marinc., M.J. Wingf.			Coetzee et al. 2015
						* dunense *	Tchotet, Rajchenb. & Jol. Roux			Tchotet et al. 2018
						* eickeri *	Tchotet, M.P.A. Coetzee, Rachjenb. & Jol. Roux			Tchotet et al. 2019
						* eminii *	Henn.	PREM		
						*cf. cupreum*	(Sacc.) Bres			Tchotet et al. 2019
						* enigmaticum *	M.P.A. Coetzee, Marinc., M.J. Wingf.			Coetzee et al. 2015
						* fulvellum *	Bres.	PREM		
						* hildebrandii *	Henn.	PREM		
						* knysnamense *	Tchotet, M.P.A. Coetzee, Rachjenb. & Jol. Roux			Tchotet et al. 2019
						* lucidum *	(Curtis) P. Karst.	PREM	Yes	
						* mastoporum *	(Lév.) Pat.	PREM		
						* mollicarnosum *	(Lloyd) Sacc. & Trotter	PREM		
						* nigrolucidum *	(Lloyd) D.A. Reid	PREM		
						* oerstedii *	(Fr.) Torrend	PREM		
						* oregonense *	Murrill	PREM		
						* oroflavum *	(Lloyd) C.J. Humphrey	PREM		
						* resinaceum *	Boud.	PREM	Yes	Tchotet et al. 2019
						* tornatum *	(Pers.) Bres.	PREM		
						* zonatum *	Murrill	PREM		
				Incertae sedis	* Crustodontia *	* chrysocreas *	(Berk. & M.A. Curtis) Hjortstam & Ryvarden		Yes	
				Meruliaceae	* Acia *	* conferta *	P.H.B. Talbot	PREM		
						* stenodon *	(Pers.) Bourdot & Galzin	PREM		
						* uda *	(Fr.) P. Karst.	PREM		
					* Aegerita *	* webberi *	H.S. Fawc	PREM		
					* Bjerkandera *	* adusta *	(Willd.) P. Karst.	PREM	Yes	
					* Cymatoderma *	* elegans *	Jungh.	PREM	Yes	
					* Gloeoporus *	* conchoides *	Mont.	PREM		
						* dichrous *	(Fr.) Bres.	PREM		
					* Irpex *	* dregeanus *	(Berk.) P.H.B. Talbot	PREM		
Fungi	Basidiomycota	Agaricomycetes	Polyporales	Meruliaceae	* Irpex *	* flavus *	(Jungh.) Kalchbr.	PREM		
						* grossus *	Kalchbr.	PREM		
						* modestus *	Berk. ex Cooke	PREM		
						* obliquus *	(Schrad.) Fr.	PREM		
						* villereus *	Berk. & Broome	PREM		
					* Laschia *	* frieseana *	(Henn.) Sacc.	PREM		
						* pustulata *	Berk. & Broome	PREM		
						* volkensii *	Bres.	PREM		
					* Merulius *	* corium *	(Pers.) Fr.	PREM		
						* gelatinosus *	Petch	PREM		
						* himantioides *	Fr.	PREM		
						* lacrymans *	(Wulfen) Schumach.	PREM		
						* molluscus *	Fr.	PREM		
						* pinastri *	(Fr.) Burt	PREM		
						* rufus *	Pers.	PREM		
						* squalidus *	Fr.	PREM		
						* tremellosus *	Schrad.	PREM		
					* Mycoleptodon *	* ochraceus *	(Pers.) Bourdot & Galzin	PREM		
					* Odontia *	* arguta *	(Fr.) Quél.	PREM		
						* bicolor *	(Alb. & Schwein.) Quél.	PREM		
						* mellea *	(Berk. & Broome) Rea	PREM		
					* Phlebia *	* strigosozonata *	(Schwein.) Lloyd	PREM		
					* Podoscypha *	* affinis *	(Berk. & M.A. Curtis) Pat.	PREM		
						* involuta *	(Klotzsch) Imazeki	PREM		
						* parvula *	(Lloyd) D.A. Reid	PREM	Yes	
				Phanerochaetaceae	* Pseudolagarobasidium *	* acaciicola *	Ginns			Wood and Ginns 2006
				Podoscyphaceae	* Abortiporus *	* biennis *	(Bull.) Singer		Yes	
				Polyporaceae	* Coriolopsis *	* lata *	(Berk.) Ryvarden	PREM		
						* polyzona *	(Pers.) Ryvarden	PREM	Yes	
						* strumosa *	(Fr.) Ryvarden	PREM		
					* Coriolus *	* azureus *	(Fr.) G. Cunn.	PREM		
						* obducens *	(Pers.) Bourdot & Galzin	PREM		
						* pubescens *	(Schumach.) Quél.	PREM		
						* unicolor *	(Bull.) Pat.	PREM		
						* zonatus *	(Nees) Quél.	PREM		
					* Daedaleopsis *	* confragosa *	(Bolton) J. Schröt.	PREM	Yes	
					* Favolus *	* brasiliensis *	(Fr.) Fr.	PREM		
						* europaeus *	Fr.	PREM		
Fungi	Basidiomycota	Agaricomycetes	Polyporales	Polyporaceae	* Favolus *	* friesii *	Berk. & M.A. Curtis	PREM		
						* hispidulus *	Berk. & M.A. Curtis	PREM		
						* spathulatus *	(Jungh.) Lév.	PREM	Yes	
					* Fomes *	* annosus *	(Fr.) Cooke	PREM		
						* caliginosus *	(Berk.) Cooke	PREM		
						* caryophylli *	(Racib.) Bres.	PREM		
						* conchatus *	(Pers.) Gillet	PREM		
						* connatus *	(Weinm.) Gillet	PREM		
						* fraxineus *	(Bull.) Cooke	PREM		
						* fulvus *	(Scop.) Gillet	PREM		
						* geotropus *	(Cooke) Cooke	PREM		
						* gibbosus *	(Blume & T. Nees) Sacc.	PREM		
						* gilvus *	(Schwein.) Lloyd	PREM		
						* glaucoporus *	Lloyd	PREM		
						* hornodermus *	(Mont.) Cooke	PREM		
						* kamphoeveneri *	(Fr.) Sacc.	PREM		
						* langloisii *	Murrill) Sacc. & D. Sacc.	PREM		
						* laricis *	(F. Rubel) Murrill	PREM		
						* leucophaeus *	(Mont.) Cooke	PREM		
						* lignosus *	(Klotzsch) Bres.	PREM		
						* lividus *	(Kalchbr. ex Cooke) Sacc.	PREM		
						* macgregorii *	Bres.	PREM		
						* marginatus *	(Pers.) Fr.	PREM		
						* marmoratus *	(Berk. & M.A. Curtis) Cooke	PREM		
						* melanoporus *	(Mont.) Sacc.	PREM		
						* minutulus *	Henn.	PREM		
						* pachyphloeus *	Corner	PREM		
						* pectinatus *	Lloyd	PREM		
						* pinicola *	(Sw.) Cooke	PREM		
						* putearius *	Weir	PREM		
						* ribis *	(Schumach.) Gillet	PREM		
						* rimosus *	(Berk.) Cooke	PREM		
						* robinsoniae *	(Murrill) Sacc. & Trotter	PREM		
						* roburneus *	Lázaro Ibiza	PREM		
						* roseus *	(Alb. & Schwein.) Fr.	PREM		
						* scalaris *	(Berk.) Sacc.	PREM		
						* senex *	(Nees & Mont.) Cooke	PREM		
						* sessilis *	(Murrill) Sacc. & D. Sacc.	PREM		
						* ulmarius *	(Sowerby) Gillet	PREM		
Fungi	Basidiomycota	Agaricomycetes	Polyporales	Polyporaceae	* Fomes *	* ungulatus *	Lázaro Ibiza	PREM		
						* velutinus *	Bres.	PREM		
						* yucatanensis *	(Murrill) Sacc. & D. Sacc.	PREM		
						* zambesianus *	(Lloyd) Sacc.	PREM		
						* zuluensis *	Wakef.	PREM		
					* Funalia *	* gallica *	(Fr.) Bondartsev & Singer		Yes	
						* leonina *	(Klotzsch) Pat.	PREM		
						* protea *	(Berk.) D.A. Reid		Yes	
						* trogii *	(Berk.) Bondartsev & Singer		Yes	
					* Grammothele *	* pseudomappa *	P.H.B. Talbot	PREM		
					* Heliocybe *	* sulcata *	(Berk.) Redhead & Ginns		Yes	
					* Hexagonia *	* albida *	Lloyd	PREM		
						* crinigera *	Fr.	PREM		
						* discopoda *	Pat. & Har.	PREM		
						* dregeana *	Lév.	PREM		
						* friesiana *	Speg.	PREM		
						* glabra *	(P. Beauv.) Ryvarden	PREM		
						* hirta * f. hystrix	(Cooke) O. Fidalgo	PREM		
						* pobeguinii *	Har.	PREM		
						* polygramma *	(Mont.) Fr.	PREM		
						* rigida *	Berk.	PREM		
						* speciosa *	Fr.	PREM		
						* tenuis *	(Hook.) Fr.	PREM	Yes	
						* tricolor *	Fr.	PREM		
						* zambesiana *	Torrend	PREM		
					* Lentinus *	* arcularius *	(Batsch) Zmitr	PREM	Yes	
						* bissus *	Quél.	PREM		
						* fastuosus *	Kalchbr. & MacOwan	PREM		
						* flabelliformis *	(Bolton) Fr.	PREM		
						* lecomtei *	Fr.	PREM		
						* murrayi *	Kalchbr. & MacOwan	PREM		
						* nigripes *	Fr.	PREM		
						* omphalodes * var. africanus	A. Pearson			Pearson 1950
						* sajor-caju *	(Fr.) Fr.	PREM	Yes	
						* strigosus *	Fr.	PREM	Yes	
						* stuppeus *	Klotzsch	PREM	Yes	
						* tigrinus *	(Bull.) Fr.	PREM		
						* tuber-regium *	(Fr.) Fr.	PREM		
Fungi	Basidiomycota	Agaricomycetes	Polyporales	Polyporaceae	* Lentinus *	* velutinus *	Fr.	PREM	Yes	
						* villosus *	Klotzsch	PREM	Yes	
						* zeyheri *	Berk.	PREM		
					* Lenzites *	* abietina *	(Bull.) Fr.	PREM		
						* aspera *	(Klotzsch) Fr.	PREM		
						* betulina *	(L.) Fr.	PREM	Yes	
						* guineensis *	(Afzel. ex Fr.) Fr.	PREM		
						* junghuhnii *	Lév.	PREM		
						* palisoti *	(Fr.) Fr.	PREM		
						* quercina *	(L.) P. Karst.	PREM		
						* repanda *	(Mont.) Fr.	PREM		
						* tricolor *	(Bull.) Fr.	PREM		
					* Lopharia *	* lirellosa *	Kalchbr. & MacOwan			Kalchbrenner & MacOwan 1881
						* mirabilis *	(Berk. & Broome) Pat.	PREM		
					* Lignosus *	* sacer *	(Afzel. ex Fr.) Ryvarden	PREM	Yes	
					* Microporus *	* xanthopus *	(Fr.) Kuntze	PREM	Yes	
					* Nigroporus *	* vinosus *	(Berk.) Murrill	PREM		
					* Neolentinus *	* lepideus *	(Fr.) Redhead & Ginns	PREM	Yes	
					* Panus *	* stipticus *	(Bull.) Fr.	PREM		
						* stipticus * var. farinaceus	(Schumach.) Rea	PREM		
						* stuppeus *	(Klotzsch) Pegler & R.W. Rayner	PREM		
					* Perenniporia *	* ochroleuca *	(Berk.) Ryvarden	PREM		
					* Picipes *	* badius *	(Pers.) Zmitr. & Kovalenko		Yes	
					* Phellinus *	* badius *	(Cooke) G. Cunn.	PREM	Yes	
						* robustus *	(P. Karst.) Bourdot & Galzin	PREM	Yes	
					* Polyporus *	* adustus *	(Willd.) Fr.	PREM		
						* affinis *	Blume & T. Nees	PREM		
						* anebus *	Berk.	PREM		
						* arenosobasus *	Lloyd	PREM		
						* australiensis *	Wakef.	PREM		
						* baurii *	Kalchbr.	PREM		
						* berkeleyi *	Fr.	PREM		
						* biformis *	Fr.	PREM		
						* chilensis *	Speg.	PREM		
						* cichoriaceus *	Berk.	PREM		
						* conchatus *	C.G. Loyd	PREM		
						* cotoneus *	Pat. & Har.	PREM		
						* cuticularis *	(Bull.) Fr.	PREM		
Fungi	Basidiomycota	Agaricomycetes	Polyporales	Polyporaceae	* Polyporus *	* dictyopus *	Mont.	PREM	Yes	
						* doidgeae *	Wakef.	PREM		
						* durbanensis *	Van der Byl	PREM		
						* durus *	(Timm) Kreisel	PREM		
						* favoloides *	Henn.	PREM		
						* flabelliformis *	Klotzsch	PREM		
						* flexilis *	Van der Byl	PREM		
						* fruticum *	Berk. & M.A. Curtis	PREM		
						* gilvus *	(Schwein.) Fr.	PREM		
						* grammocephalus *	Berk.	PREM		
						* heteroclitus *	(Bolton) Fr.	PREM		
						* immaculatus *	Berk. ex Lloyd	PREM		
						* isidioides *	Berk,			Berkeley 1843
						* mastoporus *	Lév.	PREM		
						* ochrolaccatus *	Mont.	PREM		
						* ochroleucus *	Berk.	PREM		
						* ochroporus *	Van der Byl	PREM		
						* patouillardi *	Lloyd	PREM		
						* picipes *	Rostk.	PREM		
						* pocula *	(Fr.) Berk. & M.A. Curtis	PREM		
						* radiatus *	(Sowerby) Fr.	PREM		
						* rhipidium *	Berk.	PREM		
						* rubidus *	Berk.	PREM		
						* rugulosus *	Lasch	PREM		
						* rusticus *	C.G. Loyd	PREM		
						* schweinitzii *	Fr.	PREM		
						* semipileatus *	Peck	PREM		
						* setiporus *	Berk.	PREM		
						* squamosus *	(Huds.) Fr.	PREM		
						* subradiatus *	Bres.	PREM		
						* telfairii *	Klotzsch	PREM		
						* trichiliae *	Van der Byl	PREM		
						* undatus *	Pers.	PREM		
						* varius *	(Pers.) Fr.	PREM		
						* vibecinus * var. antilopum	Kalchbr.	PREM		
					* Pycnoporus *	* sanguineus *	(L.) Murrill	PREM	Yes	
					* Trametes *	* albotexta *	C.G. Loyd	PREM		
						* capensis *	Lloyd			Doidge 1950
Fungi	Basidiomycota	Agaricomycetes	Polyporales	Polyporaceae	* Trametes *	* cingulata *	Berk.		Yes	
						* elegans *	(Spreng.) Fr.	PREM	Yes	
						* gibbosa *	(Pers.) Fr.		Yes	
						* griseolilacina *	Van der Byl	PREM		
						* hirsuta *	(Wulfen) Loyd	PREM	Yes	
						* keetii *	Van der Byl	PREM		
						* meyenii *	(Klotzsch) Lloyd		Yes	
						* subflava *	C.G. Loyd	PREM		
						* versicolor *	(L.) Loyd	PREM	Yes	
			Russulales	Auriscalpiaceae	* Lentinellus *	* omphalodes * var. africanus	A. Pearson		Yes	
				Hericiaceae	* Dentipellicula *	* austroafricana *	Jia J. Chen, L.L. Shen & Y.C. Dai			Chen et al. 2015
					* Laxitextum *	* bicolor *	(Pers.) Lentz	PREM	Yes	
				Lachnocladiaceae	* Asterostroma *	* cervicolor *	(Berk. & M.A. Curtis) Massee	PREM		
					* Dichostereum *	* rhodosporum *	(Wakef.) Boidin & Lanq.	PREM		
					* Lachnocladium *	* cristatum *	Lloyd	PREM		
						* zenkeri *	Henn.	PREM		
				Peniophoraceae	* Peniophora *	* arenata *	P.H.B. Talbot	PREM		
						* aspera *	(Pers.) Sacc.	PREM		
						* carnea *	(Willd.) P. Karst.	PREM		
						* cinerea *	(Pers.) Cooke	PREM		
						* filamentosa *	(Berk. & M.A. Curtis) Moffatt	PREM		
						* gigantea *	(Fr.) Massee	PREM		
						* heterocystidia *	Burt	PREM		
						* longispora * var. brachyspora	P.H.B. Talbot & V.C. Green	PREM		
						* lycii *	Höhn. & Litsch.	PREM		
						* pelliculosa *	P.H.B. Talbot	PREM		
						* quercina *	(Pers.) Cooke	PREM		
						* rimicola *	(P. Karst.) Höhn. & Litsch.	PREM		
						* roumeguerei *	(Bres.) Bres.	PREM		
						* tenuis *	(Pat.) Massee	PREM		
						* tristicula *	(Berk. & Broome) Boidin & Lanq.	PREM		
						* velutina *	(DC.) Cooke	PREM		
				Russulaceae	* Lactarius *	* deliciosus *	(L.) Gray	PREM	Yes	
						* hepaticus *	Plowr.	PREM	Yes	
					* Lactifluus *	* piperatus *	(L.) Pers.	PREM		Van der Westhuizen and Eicker 1988
					* Russula *	* agaricina *	(Kalchbr. ex Berk.) Trappe & T.F. Elliott			Berkeley 1876
						* caerulea *	Fr.		Yes	
Fungi	Basidiomycota	Agaricomycetes	Russulales	Russulaceae	* Russula *	* capensis *	A. Pearson		Yes	
						* fallax *	(Fr.) Fr.		Yes	
						* sardonia *	Fr.	PREM	Yes	
						* sororia *	(Fr.) Romell		Yes	
						* xerampelina *	(Schaeff.) Fr.		Yes	
				Stereaceae	* Aleurodiscus *	* acerinus * var. longispora	Höhn. & Litsch.,	PREM		
						* disciformis *	(DC.) Pat.	PREM		
						* limonisporus *	D.A. Reid	PREM		
						* mirabilis *	(Berk. & M.A. Curtis) Höhn.	PREM		
						* polygonioides *	(P. Karst.) Pilát	PREM		
						* roseus *	(Pers.) Höhn. & Litsch.	PREM		
					* Stereum *	* adnatum *	C.G. Loyd	PREM		
						* australe *	Lloyd		Yes	
						* erumpens *	Burt			Burt 1920
						* hirsutum *	(Wild.) Pers.	PREM	Yes	
						* laxum *	C.G. Loyd	PREM		
						* ostrea *	(Blume & T. Nees) Fr.		Yes	
						* rimosum * var. africanum	P.H.B. Talbot	PREM		
						* subpiliatum *	Berk. & M.A. Curtis	PREM		
						* tomentosum *	Van der Byl	PREM		
						* turgidum *	C.G. Loyd	PREM		
			Thelephorales	Thelephoraceae	* Hypochnus *	* eylesii *	Van der Byl	PREM		
						* michelianus *	Caldesi	PREM		
					* Thelephora *	* penicillata *	C.G. Loyd	PREM		
						* terrestris *	Ehrh.		Yes	
		Dacrymycetes	Dacrymycetales	Dacrymycetaceae	* Arrhytidia *	* involuta *	(Schwein.) Coker	PREM		
					* Calocera *	* cornea *	(Batsch) Fr.	PREM	Yes	
						* viscosa *	(Pers.) Fr.	PREM		
					* Dacrymyces *	* deliquescens *	(Bull.) Duby	PREM		
						* palmatus *	(Schwein.) Burt	PREM		
					* Dacryopinax *	* elegans *	(Berk. & M.A. Curtis) G.W. Martin	PREM		
						* spathularia *	(Schwein.) G.W. Martin	PREM	Yes	
					* Femsjonia *	* natalensis *	Cooke	PREM		
		Incertae sedis	Incertae sedis	Incertae sedis	* Naematoloma *	* capnoides *	(Fr.) P. Karst.	PREM		
						* fasciculare *	(Huds.) P. Karst.	PREM		
		Tremellomycetes	Tremellales	Tremellaceae	* Tremella *	* fuciformis *	Berk.		Yes	
						* mesenterica *	(Schaeff.) Retz.		Yes	
Fungi	Basidiomycota	Tremellomycetes	Tremellales	Tremellaceae	* Tremella *	* micropera *	Kalchbr. & Cooke			Kalchbrenner and Cooke 1880
						* microspora *	Lloyd			Lloyd 1920
					* Phaeotremella *	* foliaceae *	(Pers.) Wedin, J.C. Zamora & Millanes		Yes	
				Sirobasidiaceae	* Sirobasidium *	* magnum *	Boedijn 1934		Yes	
	Mucoromycota	Thelephoraceae	Mucorales	Pilobolacecae	* Pilobolus *	* crystallinus *	(F.H. Wigg.) Tode		Yes	
Amoebozoa	Mycetozoa	Myxomycetes	Echinosteliales	Echinosteliaceae	* Echinostelium *	* coelocephalum *	T.E.Brooks & H.W.Keller			See text
			Liceales	Cribrariaceae	* Cribraria *	* argillacea *	(Pers. ex J.F.Gmel.) Pers.			See text
						* cancellata *	(Batsch) Nann.-Bremek.			See text
						* intricata *	Schrad.			See text
						* tenella *	Schrad.			See text
				Dictydiaethaliaceae	* Dictydiaethalium *	* plumbeum *	(Schumach.) Rostaf.			See text
				Liceaceae	* Licea *	* biforis *	Morgan			See text
						* kleistobolus *	G.W.Martin			See text
						* pedicellata *	(H.C.Gilbert) H.C.Gilbert			See text
				Tubiferaceae	* Lycogala *	* epidendrum *	(L.) Fr.			See text
						* flavofuscum *	(Ehrenb.) Rostaf.			See text
					* Reticularia *	* lycoperdon *	Bull.			See text
					* Tubifera *	* ferruginosa *	(Batsch) J.F. Gmel.			See text
			Physarales	Didymiaceae	* Diachea *	* leucopodia *	(Bull.) Rostaf.			See text
					* Diderma *	* subdictyospermum *	(Rostaf.) G.Lister			See text
						* effusum *	(Schwein.) Morgan			See text
						* hemisphaericum *	(Bull.) Hornem.			See text
						* saundersii *	(Berk. & Broome ex Massee) Lado			See text
					* Didymium *	* melanospermum *	(Pers.) T.Macbr.			See text
						* difforme *	(Pers.) Gray			See text
						* eximium *	Peck			See text
						* iridis *	(Ditmar) Fr.			See text
						* nigripes *	(Link) Fr.			See text
						* squamulosum *	(Alb. & Schwein.) Fr. & Palmquist			See text
					* Mucilago *	* crustacea *	F.H.Wigg.			See text
				Physaraceae	* Badhamia *	* foliicola *	Lister			See text
						* macrocarpa *	(Ces.) Rostaf.			See text
						* affinis *	Rostaf.			See text
						* nitens *	Berk.			See text
						* spinispora *	(Eliasson & N.Lundq.) H.W.Keller & Schokn.			See text
						* utricularis *	(Bull.) Berk.			See text
					* Badhamiopsis *	* ainoae *	(Yamash.) T.E.Brooks & H.W.Keller			See text
Amoebozoa	Mycetozoa	Myxomycetes	Physarales	Physaraceae	* Craterium *	* leucocephalum *	(Pers. ex J.F.Gmel.) Ditmar			See text
						* aureum *	(Schumach.) Rostaf.			See text
						* dictyosporum *	(Rostaf.) H.Neubert, Nowotny & K.Baumann			See text
						* minutum *	(Leers) Fr.			See text
					* Fuligo *	* cinerea *	(Schwein.) Morgan			See text
						* muscorum *	Alb & Schwein			See text
						* septica *	(L.) F.H.Wigg.			See text
					* Leocarpus *	* fragilis *	(Dicks.) Rostaf.			See text
					* Physarella *	* oblonga *	(Berk. & M.A.Curtis) Morgan			See text
					* Physarum *	* cinereum *	(Batsch) Pers.			See text
						* melleum *	(Berk. & Broome) Massee			See text
						* pezizoideum *	(Jungh.) Pavill. & Lagarde			See text
						* album *	(Bull.) Chevall.		Yes	See text
						* auriscalpium *	Cooke			See text
						* bitectum *	G.Lister			See text
						* bivalve *	Pers.			See text
						* bogoriense *	Racib.			See text
						* citrinum *	Schumach.			See text
						* compressum *	Alb. & Schwein.			See text
						* confertum *	T.Macbr.			See text
						* didermoides *	(Pers.) Rostaf.			See text
						* digitatum *	G.Lister & Farquharson		Yes	See text
						* flavicomum *	Berk.			See text
						* gyrosum *	Rostaf.			See text
						* javanicum *	Racib.			See text
						* leucophaeum *	Fr.			See text
						* leucopus *	Link			See text
						* mutabile *	(Rostaf.) G.Lister			See text
						* notabile *	T.Macbr.			See text
						* nucleatum *	Rex			See text
						* penetrale *	Rex			See text
						* pusillum *	(Berk. & M.A.Curtis) G.Lister			See text
						* roseum *	Berk. & Broome			See text
						* stellatum *	(Massee) G.W.Martin			See text
						* tenerum *	Rex			See text
						* vernum *	Sommerf.			See text
						* viride *	(Bull.) Pers.			See text
					* Willkommlangea *	* reticulata *	(Alb. & Schwein.) Kuntze			See text
Amoebozoa	Mycetozoa	Myxomycetes	Stemonitales	Stemonitidaceae	* Amaurochaete *	* atra *	(Alb. & Schwein.) Rostaf.			See text
					* Comatricha *	* alta *	Preuss			See text
						* nigra *	(Pers. ex J.F.Gmel.) J.Schröt.			See text
					* Enerthenema *	* papillatum *	(Pers.) Rostaf.			See text
					* Lamproderma *	* arcyrioides *	(Sommerf.) Rostaf.			See text
						* scintillans *	(Berk. & Broome) Morgan			See text
					* Stemonaria *	* irregularis *	(Rex) Nann.-Bremek., R.Sharma & Y.Yamam.			See text
						* longa *	(Peck) Nann.-Bremek.			See text
					* Stemonitis *	* splendens *	Rostaf.			See text
						* axifera *	(Bull.) T.Macbr.			See text
						* fusca *	Roth			See text
						* herbatica *	Peck			See text
						* pallida *	Wingate			See text
					* Stemonitopsis *	* typhina *	(F.H.Wigg.) Nann.-Bremek.			See text
			Trichiales	Dianemaceae	* Calomyxa *	* metallica *	(Berk.) Nieuwl.			See text
				Trichiaceae	* Arcyria *	* cinerea *	(Bull.) Pers.			See text
						* denudata *	(L.) Wettst.			See text
						* incarnata *	(Pers. ex J.F.Gmel.) Pers.			See text
						* insignis *	Kalchbr. & Cooke			See text
						* minuta *	Buchet			See text
						* obvelata *	(Oeder) Onsberg			See text
						* oerstedii *	Rostaf.			See text
						* pomiformis *	(Leers) Rostaf.			See text
					* Hemitrichia *	* clavata *	(Pers.) Rostaf.			See text
						* serpula *	(Scop.) Rostaf. ex Lister			See text
					* Metatrichia *	* vesparia *	(Batsch) Nann.-Bremek. ex G.W.Martin & Alexop.			See text
					* Oligonema *	* schweinitzii *	(Berk.) G.W.Martin			See text
					* Perichaena *	* depressa *	Lib.			See text
						* corticalis *	(Batsch) Rostaf.			See text
					* Trichia *	* persimilis *	P.Karst.			See text
						* affinis *	de Bary			See text
						* botrytis *	(J.F.Gmel.) Pers.			See text
						* favoginea *	(Batsch) Pers.			See text
						* scabra *	Rostaf.			See text
						* varia *	(Pers. ex J.F.Gmel.) Pers.			See text
		Protostelids	Protosteliales	Ceratiomyxaceae	* Ceratiomyxa *	* fruticulosa *	(O.F.Müll.) T.Macbr.			See text
					* Ceratium *	* sphaeroideum *	Kalchbr. & Cooke			Kalchbrenner and Cooke 1880

**Figure 1.**
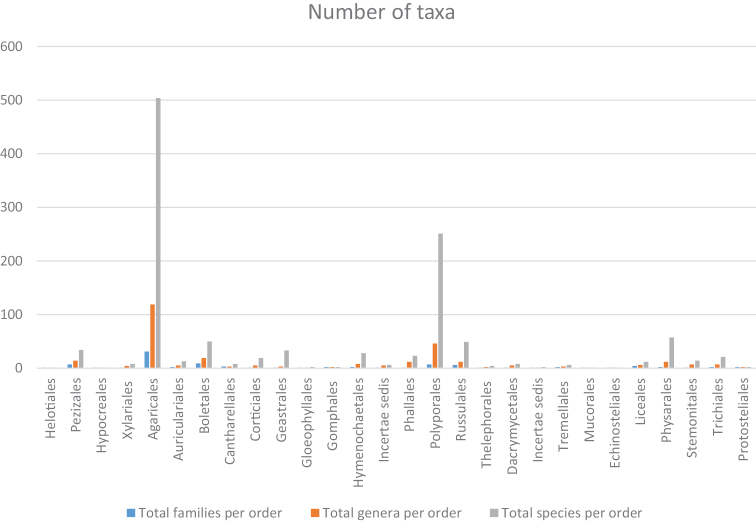
Bargraph indicating proportions of families, genera and species per order.

The Basidiomycota consisted of 1008 species, 251 genera and 72 families. At the class level, the Agaricomycetes had the highest number (Fig. 1) of species (992), genera (242), and families (68) hosting 86% of the total number of species of macrofungi. The largest order was represented by the Agaricales (504 species) followed by the Polyporales (251 species), Boletales (50 species), Russulales (49 species) and Geastrales (33 species). The smallest orders were the Gloeophyllales and Gomphales with only two species. The largest family was the Agaricaceae (180 species) followed by the Polyporaceae (172 species). Orders with only two species were the Gloeophyllale and Gomphales, while the Thelephorales had four, and the Tremmelales 6 species.

The Ascomycota was represented by 44 species distributed among 20 genera and 10 families. The Pezizomycetes had 34 species, Sordariomycetes 9 species and the Leotiomycetes one species (Table 1). However, the total number of species in these groups are biased in this study to include only those that can be considered as a macrofungus. One member of the Mucorales (*Pilobolus crystallinus*, Mucoromycota) was also included (Table 1).

More than hundred slime molds have been recorded from South Africa based on the list (Table 1, Fig. 1), with the Physarales (Myxomycota) having the most species (57 species). The 107 names of slime molds contributed originated from published and unpublished sources (Duthie 1917a, b; Doidge 1950; Ndiritu et. al. 2009; Ndiritu and De Haan 2017; Winset KE unpubl. data). Only accepted taxonomic names following the nomenclatural criteria proposed by Lado (2005–2018), which is recognized by the Encyclopedia of Life under Species 2000 and ITIS Catalogue of Life (http://eol.org/), were used. All of the five orders of myxomycetes are present in South Africa, and include the Echinosteliales (represented by one family and one genus), Liceales (four families and six genera), Physarales (two families and three genera), Stemonitales (one family and seven genera) and Trichiales (two families and seven genera). The Protosteliales (Protostelids), a sixth order though not generally regarded as myxomycetes, is represented by only two species, *Ceratiomyxa fruticulosa* (O.F. Müll.) T. Macbr and *Ceratium sphaeroideum* Kalchbr. & Cooke (Kalchbrenner and Cooke 1880; Spiegel et al. 2017).

As expected, cosmopolitan and widespread species in Africa have been reported from South Africa, such as *Arcyria cinerea* (Bull.) Pers., *Arcyria denudata* (L.) Wettst., *Stemonitis fusca* Roth, *Physarum pusillum* (Berk. & M.A. Curtis) G. Lister, *P. compressum* Alb. & Schwein., *Lycogala epidendrum* (L.) Fr., *Diderma hemisphaericum* (Bull.) Hornem., *Stemonitis splendens* Rostaf., *Didymium squamulosum* (Alb. & Schwein.) Fr. & Palmquist, *Fuligo septic* (L.) F.H. Wigg., *Hemitrichia serpula* (Scop.) Rostaf. ex Lister, *Metatrichia vesparia* (Batsch) Nann.-Bremek. ex G.W. Martin & Alexop. and *Pericheana depressa* Lib (http://www.discoverlife.org/). The number of species reported from South Africa also compares favorably with the approximately 375 myxomycete species reported from the African continent and its territories (Ndiritu and de Haan 2017). However, this is fewer than the 431 species reported from 30 countries in the Neotropics and 880 species from across the world (Lado 2005–2018).

Myxomycetes are not represented in PREM. This large deficit is most likely because slime molds have a different biology from fungi. This also reflects the limited focus that the broad fungal diversity has received in South Africa, with a much larger focus on disease causing fungi of plants, animals and humans. Even within mycological circles, slime molds have received very limited attention and there has been no expertise in studying them.

## Discussion

The checklist gives an overview of the visible mycobiota of South Africa from different sources of data. The checklist presented in this publication is the first for South African macrofungi and is as comprehensive as can be of currently collected and published macrofungi. The list will serve as a foundation to add names to a future real-time, developing, online list that should eventually become as complete as possible, similar to what is available for other organisms in South Africa such as plants and animals. Information on South African macrofungi is still scanty and a great degree of inventorying is needed to document existing species, as well as new species, in order to produce more detailed checklists of macrofungi of South Africa. It will also need future refinement and additions are already forthcoming, including ecological and distribution information.

South Africa has a long history of mycology. Based on what was published in the available field guides on macrofungi for South Africa (Stephens and Kidd 1953a, b; Levin et al. 1985; Van der Westhuizen and Eicker 1994; Branch 2001; Gryzenhout 2010; Goldman and Gryzenhout 2019), the most common macrofungal species reported across all the years belonged to several genera, including *Agaricus* L., *Amanita* Pers., *Boletus* L., *Coprinus* Pers., *Lactarius* Pers., *Laetiporus* Murr., *Macrolepiota* Singer, *Russula* Pers., and *Suillus* Gray. There exists a level of overlap of species mentioned in the different field guides, but each field guide also included unique species while not one of them is complete or comprehensive due to publishing constraints. However, even the guides combined do not yet encompass the diversity of known and unknown species present in South Africa.

A number of scientific publications exist that listed macrofungi for South Africa in general. Doidge (1950) summarized the content of her book in tabular form, listing 835 Ascomycete species, 1704 Basidiomycetes species (36%) and several species of myxomycetes. The phytopathogenic component of these species, and species discovered since then, were summarized by Crous et al. (2000). Van der Westhuizen and Eicker (1988) listed the various fungi known at that stage in the Pretoria area (Gauteng Province), while Gorter and Eicker (1988) provided Afrikaans names for a list of fungi. Vellinga et al. (2009) and Wood (2017) listed a number of fungi, including macrofungi that they considered to be introduced into South Africa.

Eicker and Baxter (1999) presented a good overview of research done on basidiomycetes from 1977 to 1999. Their publication provides references to studies on the genera and species of *Phaeolus* (Pat.) Pat., *Pisolithus* Alb. & Schwein., *Termitomyces* R. Heim, *Amanita* Pers., *Chlorophyllum* Massee, *Clathrus* P. Micheli ex L., *Hymenagaricus* Heinem., *Lepiota* (Pers.) Gray, *Macrolepiota* Singer, *Leucoagaricus* Locq. ex Singer, *Leucocoprinus* Pat., *Montagnea* Fr. and *Hymenochaete* Lév. A monograph on resupinate and stereoid *Hymenomycetes*, a revision of *Hymenochaete* Lév. (*Hymenochaetaceae*) (Job, 1987) and a series of papers dealing with *Stereum* Pers., *Lopharia* Kalchbr. & MacOwan, *Cymatoderma* Jungh. and the *Thelephoraceae* (Gorter, 1979). Paul A. van der Byl was known for his pioneering work on polypores or bracket fungi while Averil M. Bottomley documented South African Gasteromycetes (Bottomley, 1948). New species of Gasteromycetes were described, such as *Bovista acocksii* De Villiers, Eicker & Van der Westhuizen (De Villiers et al. 1989), but limited information is still available for the Geasteraceae of South Africa (Coetzee and Van Wyk 2003). A new basidiomycetous species, namely *Pseudolagarobasidium acaciicola* Ginns, was considered to be a potential biocontrol against the invasive weed *Acacia cyclops* (Wood and Ginns 2006; Kotzé et al. 2015).

A number of recent studies on macrofungi included DNA phylogenetic data. For example, studies during the early part of the last century reported *Armillaria mellea* (Vahl: Fr.) P. Kumm. in South Africa (Pole 1933; Kotzé 1935; Bottomley 1937), that was largely associated with an expanding plantation forestry industry and the pathogenic nature of the fungus. However, recent morphologic and DNA-based studies showed that the fungus killing pine trees in South Africa is *A. fuscipes* Petch (Coetzee et al. 2000), while the Northern Hemisphere species *A. mellea* and *A. gallica* Marxm. & Romagn. are restricted to the Western Cape on non-native trees and dying *Protea* plants in the Kirstenbosch Botanical Gardens, respectively (Coetzee et al. 2000, 2003). However, recent studies alarmingly showed that *A. mellea* is spreading to native fynbos areas and is able to infect a number of native plants in natural ecosystems of the Western Cape (Coetzee et al. 2018).

A number of new *Ganoderma* species were discovered through the use of DNA sequences. These include *Ganoderma austroafricanum* Coetzee, M.J. Wingf., Marinc., Blanchette on *Jacaranda mimosifolia*, which was assumed to be the main causal agent of root rot on these trees (Crous et al. 2014), *G. enigmaticum* M.P.A. Coetzee, Marinc., M.J. Wingf. and *G. destructans* M.P.A. Coetzee, Marinc., M.J. Wingf. (Coetzee et al. 2015). *Ganoderma destructans*, another novel species *G. dunense* Tchotet, Rachjenb. & Jol. Roux, an undescribed novel species of *Ganoderma*, and *Pseudolagaricobasidion acaciicola* were also found associated with dying plants of the invasive weed *Acacia cyclops* in the Eastern and Western Cape Province (Tchoumi et al. 2018). A survey (Tchotet et al. 2017) on wood-rotting basidiomycetes from various declining native tree species in the Garden Route National Park (Western Cape) also showed *Ganoderma* to be the most prominent associated group, together with *Innonotus*, *Fomitoparia* and *Wrightoporia* to a lesser degree. The study also defined other operational taxonomic units (OTUs) with sequence data from such symptoms, and assigned tentative identities based on closest sequence hits on the UNITE database. In Tchotet et al. (2019) the OTU’s belonging to *Ganoderma* was further characterized based on multi-gene phylogenies and brought up the number of *Ganoderma* species present in South Africa to 13. From the study another two new species, namely *G. eickeri* Tchotet, M.P.A. Coetzee, Rachjenb. & Jol. Roux and *G. knysnamense* Tchotet, M.P.A. Coetzee, Rachjenb. & Jol. Roux, were described, and the two phylogenetetic groups named as G. cf. resinaceum Boud. and G. cf. cupreum (Sacc.) Bres. could indicate the first reports of these species in South Africa. Ganoderma cf. cupreum has not been previously collected or observed (Table 1), while specimens of *G. resinaceum* are present in PREM and the species has been recorded previously (Table 1).

A new *Fomitiporia* species, *F. capensis* M. Fisch., M. Cloete, L. Mostert, F. Halleen, was described from South Africa based on fruit body morphology and combined internal transcribed spacer (ITS) and large-subunit ribosomal RNA gene (LSU) sequence comparisons (Cloete et al. 2014). The new species *Phellinus resupinatus* M. Fisch., M. Cloete, L. Mostert, F. Halleen, was found to be associated with the disease esca and white rot on grape vines (Cloete et al. 2016). Two new *Chlorophyllum* species, namely *C. palaeotropicum* Z.W. Ge & A. Jacobs and *C. africanum* Z.W. Ge & A. Jacobs, were described based on morphology and DNA sequences of the ITS, partial LSU, the second largest subunit of RNA polymerase II (*rpb*2) and translation elongation factor 1-α (*tef*1) sequences (Ge et al. 2018). The jacaranda stinkhorn (*Itajahya galericulata* Möller) in Pretoria was also typed phylogenetically (Marincowitz et al. 2015).

Fungi associated with termite mounds formed the focus of a number of studies. *Termitomyces* spp. associated with some termite species are arguably some of the best known fungi among non-specialists in South Africa, as they are rather obvious, numerous, interesting and a well-loved delicacy. A number of species have been described from South Africa (Botha and Eicker 1991a, b; Eicker and Baxter 1999; Fine Licht et al. 2005), but not all species of *Termitomyces* associated with the 42 South African fungus growing termite species have been characterized. Neither have the *Xylaria* Hill ex Schrank species (Ascomycetes, Xylariaceae) associated with termite nests been fully characterized. However, *X. fioriana* Sacc. was identified and described in South Africa (Saccardo 1891). Another well-known associate with termite mounds, *Podaxis pistillaris* (L.) Fr., was also found to consist of more than one phylogenetic lineage, including several collections from Africa, that could be supported morphologically and ecologically (Conlon et al. 2016, 2019).

A total of 105 myxomycete species (Table 1) are known from South Africa (Ndiritu and De Haan 2017). The first record of myxomycetes of South African myxomycetes was published in 1917 (Duthie 1917a). Additional published surveys included Duthie (1917b) and Doidge (1950). One would expect more species in South Africa especially when considering the presence of diverse habitats across such a large surface area. Clearly, this is a vastly understudied and underexploited group in South Africa supported by no local expertise.

A number of species presented in past field guides (Table 1), which should present studied fungi, do not have specimens lodged in PREM (15%, excluding slime molds) and are thus not present in our National Collection. For instance, none of the important termite-cultivated *Termitomyces* species, including the iconic *Termitomyces umkowaan* (Cooke & Massee) D.A. Reid that is readily consumed by many, has fungorium specimens in PREM. These even include commonly occurring species such as *Schizophyllum commune* Fr. that are widespread throughout South Africa and that can even be observed in dry conditions. Only 14% of fungi (excluding slime molds) published in previous field guides are also lodged in PREM (Table 1).

Conversely, a very large proportion of species in PREM (77% excluding slime molds) have not been included in popular field guides and are thus largely unknown to citizens interested in these fungi, and even professional mycologists. These pieces of forgotten knowledge are crucial to complete the current and future status of our fungal biodiversity, and represent a glimpse of the diversity in earlier times. For instance, 11 species of *Pholiota* (Fr.) P. Kumm. are lodged in PREM but did not feature in previous field guides. A twelfth species, *P. squarrosa* (Oeder) P. Kumm., is the only species currently listed in field guides but specimens for this species are not lodged in PREM (Table 1). Many of these collections representing genera or closely related groups, however, represent invaluable research opportunities to update the status of species in South Africa in the form of monographs and contemporary phylogenetic studies, to add new samples and possibly describe novel species.

Although great care was taken to eliminate possible synonyms present in the list, and to provide the most recent names for species listed under previous names (Index Fungorum 2019; Crous et al. 2004), a number of synonyms and previous names most likely are still present. It is impossible to continuously crosscheck the list, but errors can be rectified with future revisions for certain groups in the list that aim to eliminate these problems. It is also important to remember when using the list for research, that previous synonyms (including original published or collected names as listed in the contemporary taxonomic databases Index Fungorum and Mycobank) must also be searched.

A number of names listed in Doidge (1950) are not yet present in the list. Since a large proportion of these listed names have new combinations, it was uncertain whether the original author/-s observed them in the sense of what they are called today, or to what genus or species they were attributed to in the past. Some of these names also proved to be non-existent. Due to the importance of Doidge (1950) and the large number of names it contains, it was thus decided to rather treat the names included in Doidge (1950) separately where they can be more carefully linked to existing names and collections and their validity verified, before inclusion in the current checklist published here.

We emphasize that data obtained from publications and books were based on names only at this stage, because although published, some names were not supported by voucher collections that can be used to validate the accuracy of the included names. Even lists obtained from the fungorium, although tied to specimens, may represent misidentifications, previous synonyms or specimens not yet updated to recent systematic schemes for the particular taxon. Furthermore, a large number of macrofungi are still unnamed in South Africa, remain undiscovered, or new reports continue to be generated where discovered fungi could be identified. However, the working list presented here should form a solid foundation to revise names and add more names in future, especially if tied to certain targets or priorities matched to existing expertise and collaborations.

Having a fungal name list is invaluable. It is the first step towards compiling an atlas for macrofungi, similar to what exists for other organisms in South Africa (for example, Harrison et al. 1997). Such an atlas can also include distribution, ecological and biological data useful for diverse end users in governmental institutions, and those linked to conservation, ecology, academia and citizen science (Gryzenhout 2015). Additional products would be used to compile, for the first time, a red-list of macrofungi based on International Union for the Conservation of Nature (IUCN) criteria, and guidelines to protect them based on their biology. It will aid to identify indicator species to monitor ecological integrity and change. The residency status of macrofungi can be defined better, and species that are truly endemic, naturalized, introduced or invasive can be defined properly within each group. The need for this is already evident where fungi have been previously listed (Vellinga et al. 2009; Wood 2017) but there was no national list for comparison. In fact, one species listed in Vellinga et al. (2009), *Inocybe curvipes* P. Karst., is not present in previous publications or in PREM (Table 1). The checklist information can be used in education for the sustainable and safe use of fungal natural resources, to produce conservation awareness and regulation to protect naturally harvested species and habitats from over-harvesting (Gryzenhout et al. 2010, 2012). Lastly, the lists will be instrumental to do gap assessments from the compiled data to help identify research needs in future, for example where to focus surveys and collections, revisions, and where the greatest gaps for species descriptions exist. A list will also enable citizen scientist collaboration and participation and make the study of fungi more transparent (Gryzenhout 2015).

Human capacity should be developed in the area of mycology and biodiversity conservation. The species found in each region of South Africa is still unknown and there have not been any recent monographic works. Furthermore, a great need exists to continue revising the list, to ensure that more representatives of species are added and taxonomic revisions are undertaken and included in the list. The list should also be enabled to continue and long-term plans should be developed to ensure its sustainability.

The list presented is only based on species and specimens that could be named. A great deal of unknown taxa of macrofungi still exist. In fact, approximately 200 ‘‘unknown’’ macrofungal species of the fungorium records were left out from the list. Furthermore, approximately half of the records lodged in MushroomMap (http://adu.org.za/) represent fungi that could not be identified, whereas a great number of equally unknown fungi is posted on the Mushrooms for South Africa Facebook page (https://www.facebook.com/groups/MushroomsSouthernAfrica/), or communicated by citizen scientists (Gryzenhout 2015). This great deficit or inability to name numerous South African macrofungi is indicative of the great diversity that we have, the large proportion that are still undiscovered, unstudied, and hence under-utilized, and the paucity of human capacity to do this (South African Fungal Diversity Network 2013). Without active description and characterization, these fungi will remain in obscurity.
